# Unsaturated Coordination Oxygen in Zn─V─O Vacancy Clusters Enables Superb Zinc Storage Capability

**DOI:** 10.1002/advs.74996

**Published:** 2026-03-26

**Authors:** Yulong Chi, Fulong Li, Yangxian Wang, Longwei Li, Haolin Li, Xiaodong Shi, Xinlong Tian, Yihui Zou, Dongjiang Yang

**Affiliations:** ^1^ Institute of Micro/Nano Materials and Devices Ningbo University of Technology Ningbo Zhejiang P. R. China; ^2^ School of Environment and Geography State Key Laboratory of Bio‐fibers and Eco‐textiles Shandong Collaborative Innovation Center of Marine Biobased Fibers and Ecological Textiles Institute of Marine Biobased Materials Qingdao University Qingdao P. R. China; ^3^ School of Marine Science and Engineering State Key Laboratory of Tropic Ocean Engineering Materials and Materials Evaluation Hainan University Haikou P. R. China

**Keywords:** aqueous zinc‐ion batteries, dual zinc ion storage mechanism, unsaturated coordination oxygen atoms, Zn_3_(VO_4_)_2_

## Abstract

Vanadates have been extensively applied in aqueous zinc‐ion batteries (AZIBs) for the enlarged interlayer spacing and rich active sites induced by the pre‐intercalation of metal or ammonium ions. However, the strong electrostatic interaction between Zn^2+^ ions and guest cations always results in finite insertion ability of Zn^2+^ ions and low specific capacity. Herein, Zn_3_(VO_4_)_2_ with Zn─V─O vacancy clusters (Zn_3_(VO_4_)_2_‐V_ZVO_) is innovatively prepared as cathode material for AZIBs. Based on the ab initio molecular dynamics (AIMD) simulations and comprehensive characterizations, the Zn─V─O vacancy clusters are demonstrated to effectively capture most of free Zn^2+^ and H^+^ ions, reduce the inherent electrostatic interaction, and contribute the dominative capacity through abundant unsaturated coordination oxygen atoms. Additionally, part of adsorbed Zn^2+^ ions can reversibly intercalate Zn_3_(VO_4_)_2_‐V_ZVO_ and transform into Zn_3_V_2_O_7_(OH)_2_·2H_2_O to contribute the remaining capacity. Consequently, the optimized Zn_3_(VO_4_)_2_‐V_ZVO_ cathode delivers high reversible capacity of 410 mAh g^−1^ at 1.0 A g^−1^ and satisfying capacity retention of 94.1% after 800 cycles at 0.1 A g^−1^. This study not only reveals the formation and action mechanism of unsaturated coordination oxygen sites in Zn_3_(VO_4_)_2_‐V_ZVO_ cathode, but also offers new insight to design high‐performance cathode materials for AZIBs.

## Graphical abstract

Zn_3_(VO_4_)_2_ with Zn─V─O vacancy clusters was synthesized and innovatively applied as cathode material in aqueous Zn‐ion batteries. In‐situ XRD, HRTEM and ab initio molecular dynamics simulations unveil the double reaction mechanism of zinc ions. Adsorption of zinc ions on unsaturated coordination O and H^+^ on O in ‐VO_4_ contributes to the main capacity. It delivers 410 mAh g^−1^ at 1.0 A g^−1^ with 94.1% capacity retention over 800 cycles.

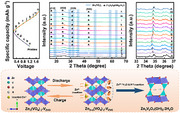

## 1 Introduction

Lithium‐ion batteries have been widely served as commercial energy storage devices for renewable energy system and portable electronics, but the large‐scale application of lithium‐ion batteries are constrained by scarce lithium resources and high manufacture cost [1, 2, 3, 4, 5]. Co tmpared with lithium, zinc holds merits of wide distribution, easy availability, low price, and nontoxicity [6, 7, 8, 9, 10, 11]. Thus, aqueous zinc‐ion batteries (AZIBs) are considered as successor of low‐cost and high‐safety energy storage system [12, 13, 14, 15, 16, 17]. Significant efforts had been made for the design of cathode materials for AZIBs, including Mn‐based compounds [18, 19], V‐based compounds [20, 21], Mo/Ni/Co‐based compounds [22, 23, 24]. In particular, V‐based compounds have been intensively studied as cathodes for AZIBs owing to the advantages of abundant resources, unique layered structure and multiple valence states of vanadium [25, 26, 27]. However, Zn^2^^+^ ions have a large ionic radius and high charge density. During the (de)intercalation process, the strong electrostatic forces between them and the main framework of V‐based compounds can easily cause lattice distortion and even lead to irreversible structural collapse [28, 29].

To solve these issues, vanadates have been extensively synthesized and used as cathodes for AZIBs [30, 31]. On the one hand, the pre‐intercalated metal or ammonium ions can effectively enlarge the interlayer distance and reduce the insertion energy barrier, thereby promoting the (de)intercalation and diffusion kinetics of Zn^2+^ ions [32]. On the other hand, the various valence states of guest ions in vanadates also can provide enough reactive sites and structural defect for the thermodynamic chemisorption of Zn^2+^ ions [33, 34, 35]. Currently, it is significant demand for vanadates to break through the following dilemmas: (1) The strong electrostatic interaction between Zn^2+^ ion and the pre‐intercalated cations results in small insertion proportion of Zn^2+^ ions; (2) The large ionic radius of raw Zn^2+^ ion and solvated Zn^2+^ ion leads to irreversible structural deformation, rapid capacity decay and even battery failure [36]. As demonstrated in previous reports [18, 37], the introduction of structure vacancy or structure defect effectively contribute to provide excess reactive sites for the adsorption/insertion of Zn^2+^ ion, furnish additional migration pathways for Zn^2+^ ions, shorten the diffusion length, and guarantee rapid diffusion kinetics as well as high structural stability, which is regarded as valid strategy to boost the zinc storage performances of vanadates. Nevertheless, there is no deep analysis about the precise action mechanism of defect/vacancy‐engineering.

In this work, ab initio molecular dynamics (AIMD) simulations and materials characterizations are performed to comprehensively reveal the zinc storage mechanism of Zn_3_(VO_4_)_2_ with Zn─V─O vacancy clusters (labeled as Zn_3_(VO_4_)_2_‐V_ZVO_). Specifically, the attendance of Zn─V─O vacancy clusters introduces abundant unsaturated coordination oxygen atoms, which contribute to capture most of drifting Zn^2+^ and H^+^ ions around the active sites of Zn_3_(VO_4_)_2_‐V_ZVO_. Density functional theory (DFT) calculations suggest the unsaturated coordination oxygen atoms can reduce the electrostatic interaction and crystal structure change of Zn_3_(VO_4_)_2_‐V_ZVO_ cathode during the intercalation process of Zn^2+^ ions. Most importantly, the fundamental effect of unsaturated coordination oxygen sites in Zn_3_(VO_4_)_2_‐V_ZVO_ on the reaction mechanism are also disclosed by the *in‐situ* characterization technique, verifying the reversible phase transformation from Zn_3_(VO_4_)_2_‐V_ZVO_ to Zn_3_V_2_O_7_(OH)_2_·2H_2_O. As expected, the home‐made Zn_3_(VO_4_)_2_‐V_ZVO_ cathode can display high reversible capacity of 410 mAh g^−1^ at 1 A g^−1^ and high‐capacity retention rate of 94.1% after 800 cycles at low current density of 0.1 A g^−1^, manifesting superior structural stability and reversibility.

## 2 Experimental Section

### 2.1 Synthesis of Zn_3_(VO_4_)_2_ and Zn_3_(VO_4_)_2_‐V_ZVO_

First, 1.5 g carboxylated chitosan and 1 g sodium alginate were dissolved in 100 mL water and stirred to form a colloidal sol. 5.85 g ammonium metavanadate and 11 g zinc acetate were dissolved in 500 mL water. Then, the colloid was added to the above mixed salt solution drop by drop. The abundant amino group in carboxylated chitosan and carboxyl group in sodium alginate could better complex zinc ions and metavanadate ions to obtain a composite gel network. The resulting composite gel was freeze‐dried to form an aerogel, and finally calcined in air atmosphere at 600°C for 2 h at a heating rate of 5°C min^−1^ to obtain Zn_3_(VO_4_)_2_.

The obtained Zn_3_(VO_4_)_2_ was impregnated in 0.5 mol/L hydrochloric acid for 0.5 h, then filtered and rinsed with distilled water until neutral. Then, Zn_3_(VO_4_)_2_ containing Zn─V─O vacancy clusters (Zn_3_(VO_4_)_2_‐V_ZVO_) was obtained. In the experiment, different concentrations of hydrochloric acid (0.1 moL/L, 0.5 moL/L, 1.0 moL/L) were used to etch Zn_3_(VO_4_)_2_, and the corresponding samples were denoted as Zn_3_(VO_4_)_2_‐0.1V_ZVO_, Zn_3_(VO_4_)_2_‐0.5V_ZVO_ and Zn_3_(VO_4_)_2_‐1.0V_ZVO_, respectively.

### 2.2 Material Characterization

X‐ray powder diffractometers (Cu Kα XRD, D8 Advance) and Fourier transform infrared spectroscopy (FTIR, Bruker VERTEX70) are used to characterize the crystalline form and structure of the as‐prepared samples. The X‐ray photoelectron spectroscopy (XPS) measurements were performed using an ESCALab250 electron spectrometer (Thermo Scientific Corporation) with monochromatic 150 W Al Kα radiation. Transmission electron microscopy (TEM) and high‐resolution TEM (HRTEM) images were obtained using a FEI Tecnai 20 TEM operating at an accelerating voltage of 200 kV. Zn K‐edge X‐ray absorption near edge structure (XANES) and extended X‐ray absorption fine structure (EXAFS) were deeply investigated on the TPS‐BL11B beamline at the National Synchrotron Radiation Research Center (NSRRC). The specific surface area of the specimens was quantified using the Brunauer‐Emmett‐Teller (BET) method for nitrogen adsorption/desorption, employing the ASAP2460 analyzer. Furthermore, the pore size distribution of the samples was computed utilizing the Barrett‐Joyner‐Halenda (BJH) model. Electron paramagnetic resonance (EPR) experiments were carried out on a Bruker ELEXSYS E500 X‐band spectrometer. All measurements were conducted at room temperature using deionized water. 10 ± 2 µL min^−1^ of water droplets were added onto the thin film surface. Contact angle measurements were performed using a contact angle measurement instrument equipped with a CCD camera. Thermogravimetric (TG) analysis was performed on the sample in N_2_ at 30°C to 800°C using a NETZSCH STA 449F3 thermogravimetric analyzer.

### 2.3 Electrochemical Characterization

Electrochemical measurements were performed on CR2025 type coin cells. A glass fiber (Whatman, GF/D) and were utilized as the separator.The electrolyte was 2.0 M solution of ZnSO_4_. The working electrode was prepared by compressing a mixture of the active materials, conductive carbon black, and binder (polyvinylidene fluoride, PVDF) in a weight ratio of Zn_3_(VO_4_)_2_ and (Zn_3_(VO_4_)_2_‐0.5V_ZVO_): carbon black: PVDF = 8:1:1 to form the slurry. The slurry was evenly pasted on the titanium foil collector and dried in a 100°C vacuum oven overnight. The diameter of the electrode sheet was 1.2 cm, the weight of the active mass was about 1.1 mg, and the mass loading was about 1 mg/cm^2^. The titanium foil was used as the collector. The cyclic voltammetry (CV), Electrochemical Impedance Spectroscopy (EIS) was tested on CHI 760E electrochemical workstation. The electrode capacity was measured by a galvanostatic charge/discharge method at a battery test system (Land CT2001A).

### 2.4 Electrochemical Kinetic Analysis

During the CV test, the peak current at different scan rates was measured, and the response between scan rate and peak current was analyzed to distinguish between diffusion and pseudocapacitive behavior during charge and discharge. This relationship can be expressed by the following empirical formula:

(1) logi(v)=loga+blogv

Both a and b are variable parameters in the formula. The b‐value is the slope of the log*i vs*. log*v* line,

When *b* = 0.5, the peak current varies with the power of 0.5 of the scan rate. The charge transfer process is a Faraday intercalation process controlled by conventional diffusion. When *b* = 1, the peak current varies linearly with the scan rate, and the energy storage process depends on the adsorption/desorption of surface charges. When *b* = 0.5∼1, this is a transition region between pseudocapacitive materials and battery‐type materials.

In a CV test, taking all factors into account, a functional relationship between current and scan rate is derived as follows:

(2) i(V)/v1/2=k1v1/2+k2

*k_1_* and *k_2_* can be calculated from the slope and intercept of the linear plot of i(V)/*v* ^1/2^ and *v* ^1/2^. In order to illustrate the favorable Zn^2+^ diffusion of Zn─V─O vacancy clusters, the diffusion coefficient of Zn^2+^ (D_Zn2+_) can also be calculated according to the following formula:

(3) $$D_{\mathrm{Zn}2+}=R^{2}\;T^{2}/2A^{2}n^{4}F^{4}C^{2}\delta^{2}$$

where R is the gas constant, T is the absolute temperature, A is the electrochemical reaction area, m: mass of active material, C is the concentration of Zn^2+^ in the electrolyte, and δ is the Warburg factor which has relationship with Z':

(4) $$Z^{\prime }=R_{D}+R_{C}+\delta \omega^{-1/2}$$

### 2.5 Density Functional Theory (DFT) Calculations

All geometric structures were optimized using the CP2k 2023.1 program package. The PBE functional in combination with Grimme's dispersion correction (D3) and Becke−Johnson damping factor (BJ) were used during geometry optimization and single point calculations. The DZVP‐MOLOPT‐SR‐GTH basis set was used during optmization and AIMD, while TZVP‐MOLOPT‐SR‐GTH during single point calculations. All analysis were carried out using the Multiwfn 3.8 Dev software package and visualized by VESTA.

## 3 Results and Discussion

**FIGURE 1:**
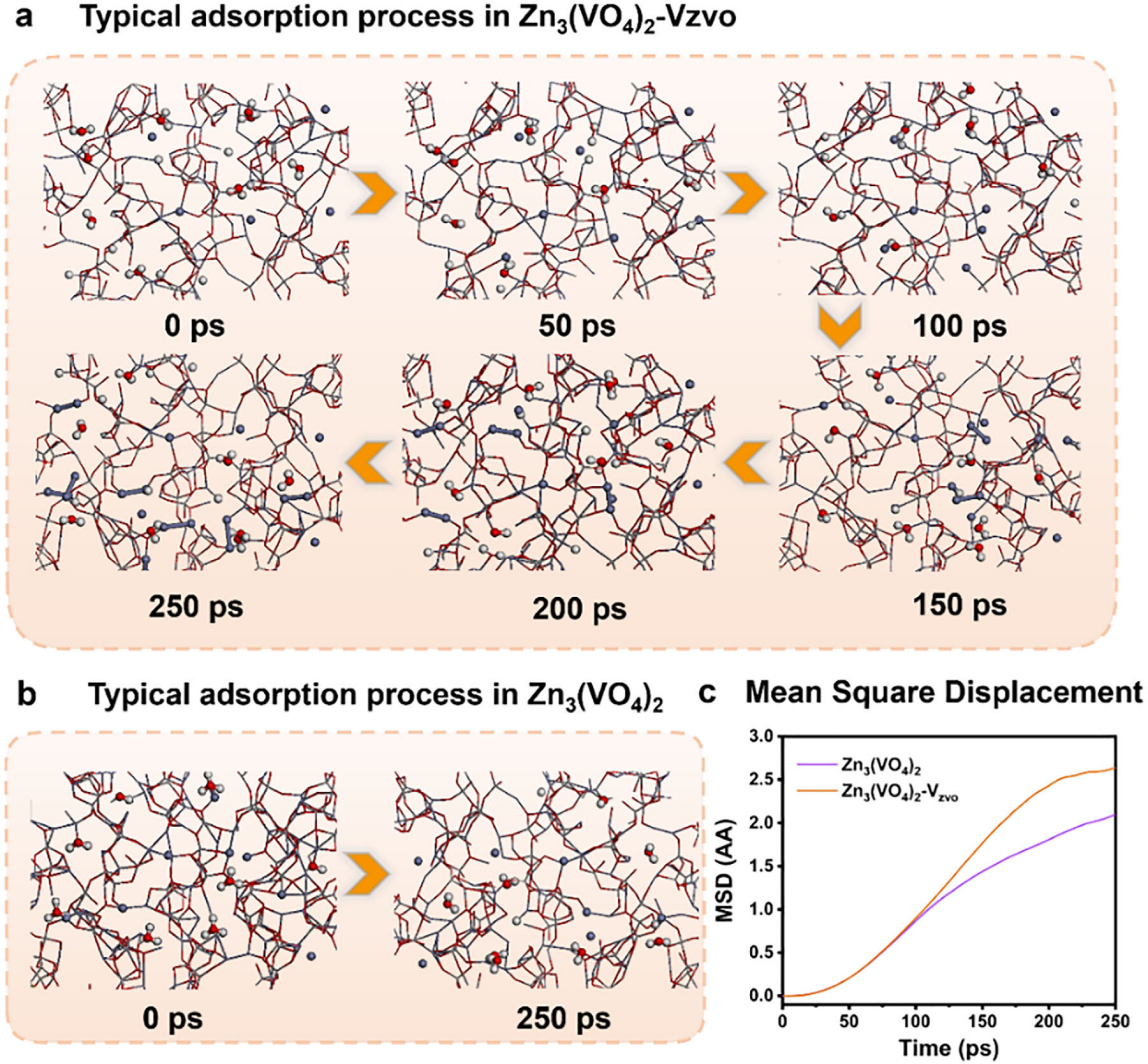
Snapshots of typical adsorption process for Zn^2+^, H^+^, OH^−^, H_2_O in (a) Zn_3_(VO_4_)_2_‐Vzvo and (b) Zn_3_(VO_4_). The red, purple, grey and white balls represent O, Zn, V, and H, respectively; (c) Mean square displacement of Zn in Zn_3_(VO_4_)_2_‐Vzvo and Zn_3_(VO_4_)_2_.

AIMD simulations are first conducted to simulate the zinc storage potential of Zn_3_(VO_4_)_2_‐V_ZVO_ and Zn_3_(VO_4_)_2_ cathode (Figure 1a; Figure S1). The snapshots at 0, 50, 100, 150, 200 and 250 ps of the AIMD process for Zn_3_(VO_4_)_2_‐V_ZVO_ are taken to plot. The vacancy clusters in Zn_3_(VO_4_)_2_‐V_ZVO_ make the end group have abundant unsaturated coordination oxygen atoms, which can capture most of drifting Zn^2+^ ions and H^+^ into Zn_3_(VO_4_)_2_‐V_ZVO_. Another small part of zinc ion could be adsorbed on ‐O‐Zn to form an ‐O‐Zn‐Zn structure. This adsorption process of Zn^2+^ and H^+^ will provide the main capacity. In addition, OH^−^ and H_2_O drifting into Zn_3_(VO_4_)_2_‐V_ZVO_ are inclined to be adsorbed on O of ‐VO_4_, which may undergo a phase transition with the adsorbed zinc ion to provide another part capacity. As shown in Figure 1b, when the simulation was carried out to 250 ps in Zn_3_(VO_4_)_2_, there are still a part of zinc ions not adsorbed on the electrode material, existing in the electrode in a free state. It is much more difficult for zinc ions to be adsorbed on Zn_3_(VO_4_)_2_ compared with Zn_3_(VO_4_)_2_‐V_ZVO_ (Figure 1a; Figure S1). In addition, vacancy clusters provide large voids, also contributing to the diffusion and adsorption of Zn. To intuitively reveal the changes of Zn diffusion in Zn_3_(VO_4_)_2_ and Zn_3_(VO_4_)_2_‐V_ZVO_, mean square displacement is performed (Figure 1c), and the higher slope of Zn_3_(VO_4_)_2_‐V_ZVO_ indicates the faster zinc migration, suggesting the vacancy clusters in Zn_3_(VO_4_)_2_‐V_ZVO_ are beneficial for the zinc storage performance.

**FIGURE 2:**
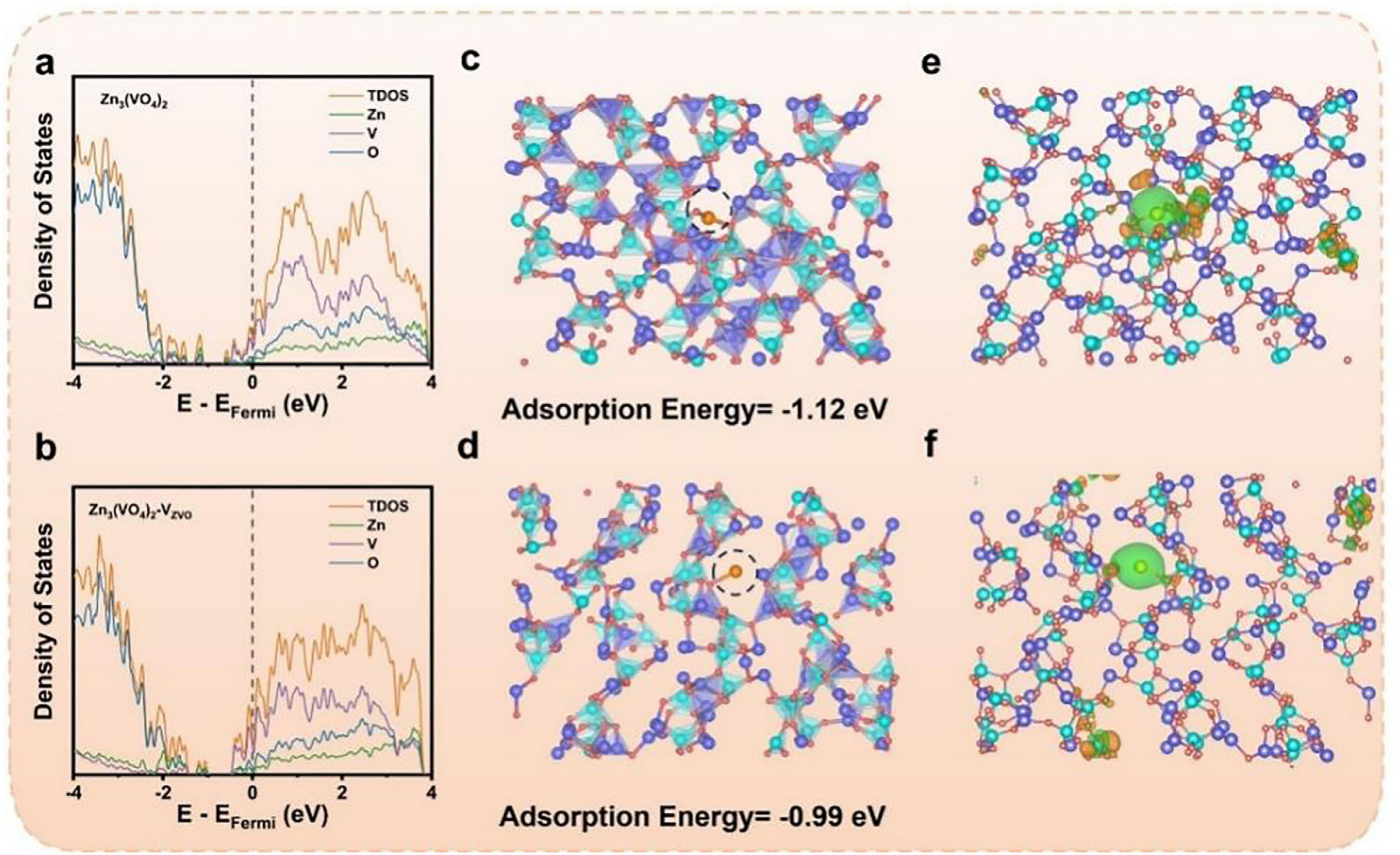
Density of states of (a) Zn_3_(VO_4_)_2_ and (b) Zn_3_(VO_4_)_2_‐Vzvo; Adsorption energy (ΔE_b_) for Zn^2+^ storage in (c) Zn_3_(VO_4_)_2_ and (d) Zn_3_(VO_4_)_2_‐Vzvo; The corresponding differential charge density after Zn^2+^ adsorption in (e) Zn_3_(VO_4_)_2_ and (f) Zn_3_(VO_4_)_2_‐Vzvo.

DFT calculations are carried out to investigate the influence of Zn─V─O vacancy clusters on zinc storage behavior. After Zn─V─O vacancy clusters introduced in Zn_3_(VO_4_)_2_, the volume increases from 7299.26 to 8740.28 Å^3^, while the enlarged cell size is conducive to the (de)intercalation of Zn^2+^ ions (Table S1). According to the calculated density of states, the bandgap of Zn_3_(VO_4_)_2_‐V_ZVO_ is less than that of Zn_3_(VO_4_)_2_, which increases the charge density of the entire Fermi energy level (Figure 2a,b). This will help to increase the carrier concentration and conductivity, resulting in the faster electrochemical reaction dynamics. Meanwhile, the band structure of Zn_3_(VO_4_)_2_‐V_ZVO_ is consecutive near the Fermi level, which demonstrates that Zn_3_(VO_4_)_2_‐V_ZVO_ is intrinsically metallic. Based on the equation ΔE_b_ = (E_total_ – E_fundus_ – E_Zn_), the Zn adsorption energy in Zn_3_(VO_4_)_2_‐V_ZVO_ and Zn_3_(VO_4_)_2_ was calculated, where E_total_, E_fundus_, and E_Zn_ are defined as the total energy of Zn adsorption fundus, fundus, and metal Zn, respectively. Zn_3_(VO_4_)_2_‐V_ZVO_ exhibits smaller adsorption energy for Zn^2+^ (‐0.99 eV) compared with Zn_3_(VO_4_)_2_ (‐1.12 eV) (Figure 2c,d). To investigate the electronic structures of Zn_3_(VO_4_)_2_ and Zn_3_(VO_4_)_2_‐V_ZVO_ after Zn^2+^ adsorption, charge density differences of Zn_3_(VO_4_)_2_ and Zn_3_(VO_4_)_2_‐V_ZVO_ adsorbed with Zn^2+^ have been calculated. The green part of the model represents charge accumulation and the yellow part is charge consumption. Compared with Zn_3_(VO_4_)_2_, the electron transfer regions are more dispersed in Zn_3_(VO_4_)_2_‐Vzvo (Figure 2e,f). This suggests the reduced electrostatic interaction between Zn^2+^ and cathode material deriving from the unsaturated coordination oxygen atoms, making the charge transfer more uniform. To investigate the structural stability, the volume changes before and after Zn^2+^ adsorption have been calculated (Figure S2), which are 1.61% for Zn_3_(VO_4_)_2_‐V_ZVO_ and 2.92% for Zn_3_(VO_4_)_2_, respectively. The smaller volume change means superior capability to maintain the structural stability.

**FIGURE 3:**
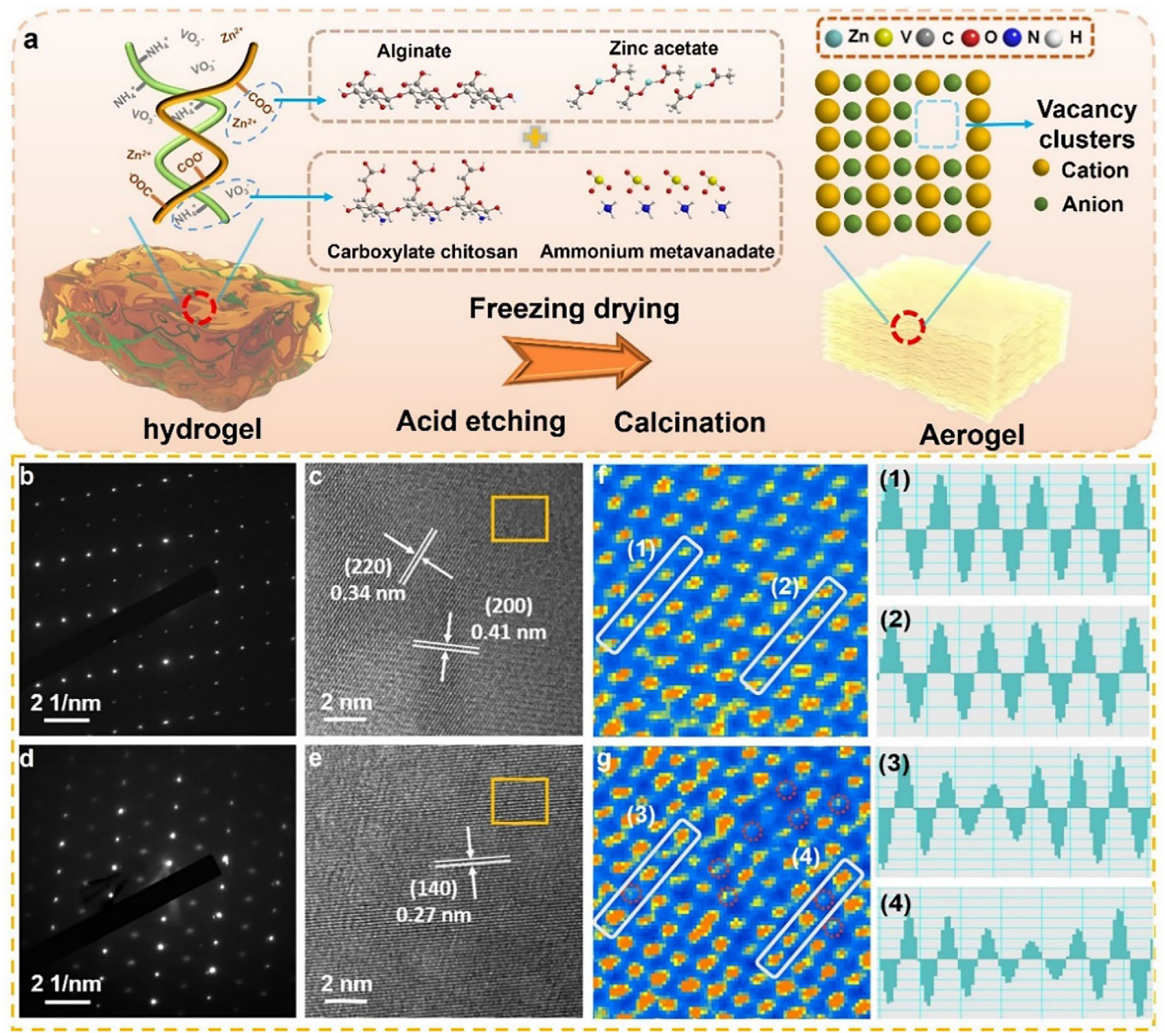
(a) Schematic illustration for the fabrication of Zn_3_(VO_4_)_2_‐0.5V_ZVO_; (b) SAED pattern and (c) HRTEM image of Zn_3_(VO_4_)_2_; (d) SAED pattern and (e) HRTEM image of Zn_3_(VO_4_)_2_‐0.5V_ZVO_; The corresponding filtered images of orange square in HRTEM images of (f) Zn_3_(VO_4_)_2_ and (g) Zn_3_(VO_4_)_2_‐0.5V_ZVO_; The corresponding normalized intensity variations in (1‐2) Zn_3_(VO_4_)_2_ and (3‐4) Zn_3_(VO_4_)_2_‐0.5V_ZVO_.

Figure 3a presents the synthesis process of Zn_3_(VO_4_)_2_ and Zn_3_(VO_4_)_2_‐V_ZVO_. First, carboxylated chitosan (CC) and sodium alginate (SA) were dissolved in distilled water and stirred to form a colloidal sol. Ammonium metavanadate and zinc acetate were dissolved in distilled water to form a mixed salt solution. Then, the colloid was added to the above mixed salt solution drop by drop. During this period, Zn^2+^ in the mixed solution chelate with the four G‐blocks of SA to form a unique “egg‐box” structure. In addition, the abundant amino group in CC could complex metavanadate ions. The resulting composite gel network with CC‐VO_4_^−^ and SA‐Zn^2+^ was then freeze‐dried to form aerogel. Second, the obtained aerogel is pyrolysed in air atmosphere to get Zn_3_(VO_4_)_2_. After then, Zn_3_(VO_4_)_2_ was impregnated in different concentrations of hydrochloric acid to get Zn_3_(VO_4_)_2_‐V_ZVO_. Zn_3_(VO_4_)_2_ and Zn_3_(VO_4_)_2_‐0.5V_ZVO_ were chosen as the representative sample to analyse the morphologies by scanning electron microscopy (SEM). As shown in Figure S3a–c, the prepared Zn_3_(VO_4_)_2_ is a micron block structure with a diameter of 1 µm. This morphology may be caused by the agglomeration of the chitosan vanadate polymer in calcination process. After hydrochloric acid etching, the surface of Zn_3_(VO_4_)_2_‐0.5V_ZVO_ presents a multi‐channel honeycomb porous structure. This structure increases the contact area between the electrode and electrolyte, promoting the rapid deintercalation of Zn^2+^. Transmission electron microscopy (TEM) and high‐resolution TEM (HRTEM) are used to investigate the evolution of vacancy clusters. Figure 3b shows a single‐crystal selected area electron diffraction (SAED) pattern, which can be indexed to Zn_3_(VO_4_)_2_ (JCPDS No. 34–0384). HRTEM image of Zn_3_(VO_4_)_2_ in Figure 3c exhibits two mutually perpendicular lattice fringes with spacing of 0.34 and 0.41 nm, corresponding to (220) and (200) lattice fringes of Zn_3_(VO_4_)_2_, respectively. Figure 3d shows the SAED pattern of Zn_3_(VO_4_)_2_‐0.5V_ZVO_, indicating that Zn_3_(VO_4_)_2_‐0.5V_ZVO_ also has a single crystal structure. HRTEM image of Zn_3_(VO_4_)_2_‐0.5V_ZVO_ (Figure 3e) shows lattice spacing of 0.27 nm, which corresponds to crystal face of (140). Figure 3f,g are the filtered images and normalized intensity changes of the white labeled regions in Zn_3_(VO_4_)_2_ and Zn_3_(VO_4_)_2_‐0.5V_ZVO_. In the normalized intensity image, the intensity distribution is directly proportional to the atomic number passing through the corresponding atomic column along the beam distribution direction. The diffraction intensity of (1) and (2) is consistent, while the diffraction intensity of (3) and (4) is unevenness, indicating that there is atom deletion in Zn_3_(VO_4_)_2_‐0.5V_ZVO_. This indicates that vacancy clusters have been produced in Zn_3_(VO_4_)_2_‐0.5V_ZVO_. In the EDS mapping image of Zn_3_(VO_4_)_2_‐0.5V_ZVO_, the overall distribution of Zn, V, O, and C elements is relatively uniform (Figure S3d–h).

**FIGURE 4:**
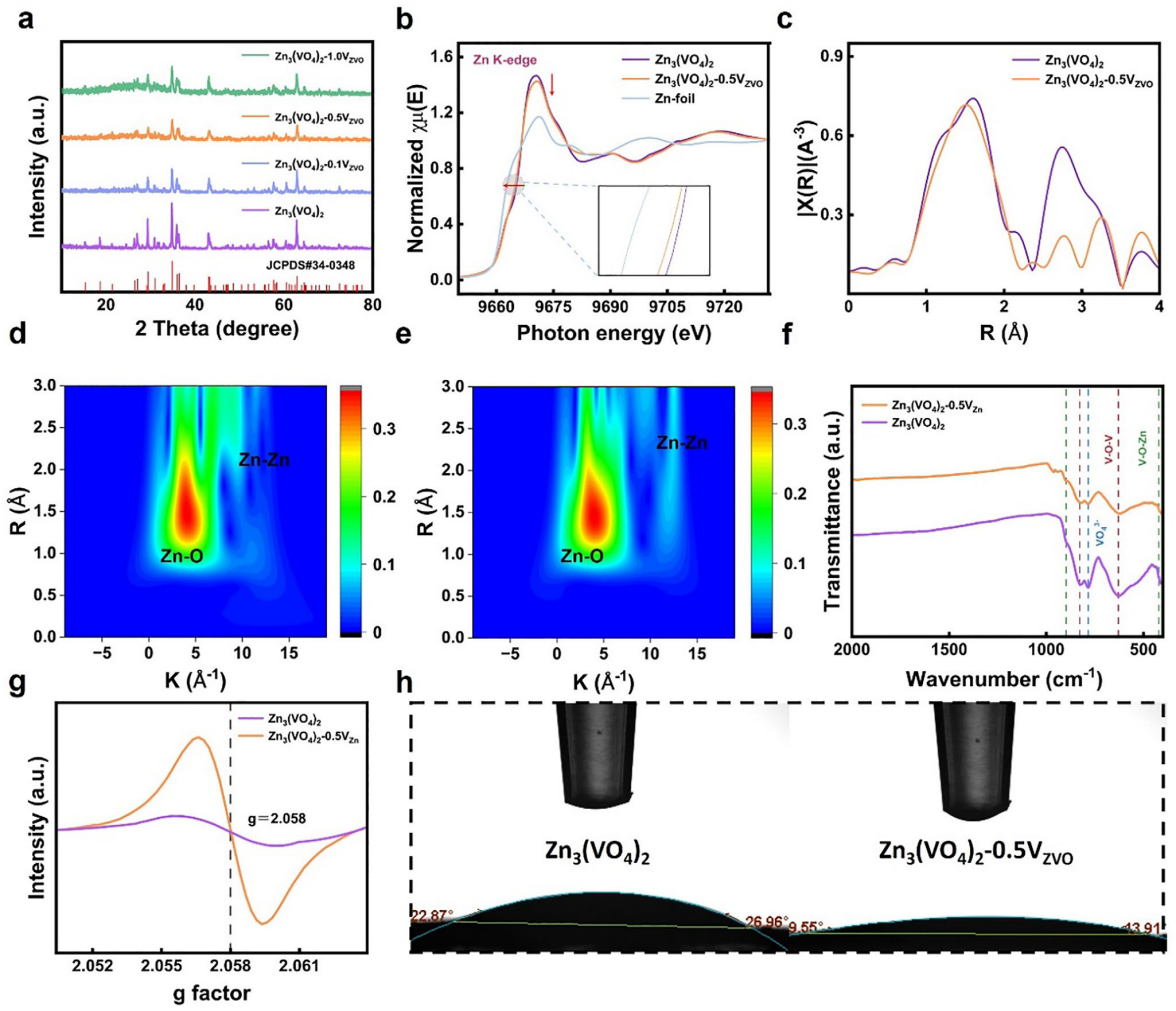
(a) XRD patterns of the Zn_3_(VO_4_)_2_, Zn_3_(VO_4_)_2_‐0.1V_ZVO_, Zn_3_(VO_4_)_2_‐0.5V_ZVO_ and Zn_3_(VO_4_)_2_‐1.0V_ZVO_; (b) Normalized XANES at the Zn K‐edge and (c) Fourier‐transform analysis of EXAFS spectra for Zn_3_(VO_4_)_2_ and Zn_3_(VO_4_)_2_‐0.5V_ZVO_; WT‐EXAFS spectra of (d) Zn_3_(VO_4_)_2_ and (e) Zn_3_(VO_4_)_2_‐0.5V_ZVO_; (f) FTIR spectra of Zn_3_(VO_4_)_2_ and Zn_3_(VO_4_)_2_‐0.5V_ZVO_; (g) EPR results of Zn_3_(VO_4_)_2_ and Zn_3_(VO_4_)_2_‐0.5V_ZVO_; (h) Contact angles of Zn_3_(VO_4_)_2_ and Zn_3_(VO_4_)_2_‐0.5V_ZVO_.

In order to analyze the structural properties of Zn_3_(VO_4_)_2_ and Zn_3_(VO_4_)_2_‐V_ZVO_, X‐ray diffraction (XRD) analysis is performed (Figure 4a). The XRD patterns show that Zn_3_(VO_4_)_2_ and Zn_3_(VO_4_)_2_‐V_ZVO_ all contain characteristic peaks corresponding to Zn_3_(VO_4_)_2_ (JCPDS 34–0348). No impurity peaks are observed in Zn_3_(VO_4_)_2_‐V_ZVO_, indicating that no phase transitions or crystal structure changes are caused by hydrochloric acid etching. To explore the formation of Zn─V─O vacancy clusters and unsaturated coordination oxygen atoms_,_ X‐ray absorption near edge spectroscopy (XANES) were performed (Figure 4b). Zn_3_(VO_4_)_2_‐0.5V_ZVO_ and Zn_3_(VO_4_)_2_ were chosen as the representative samples. The main peak of Zn_3_(VO_4_)_2_‐0.5V_ZVO_ is lower, indicating that the 1s‐3d orbital transition of Zn is reduced, confirming the existence of Zn vacancy. The Zn K‐edge XANES spectra of Zn_3_(VO_4_)_2_‐0.5V_ZVO_ show that the adsorption edge shifts to lower energies compared with that of Zn_3_(VO_4_)_2_, indicating a lower average Zn oxidation state in Zn_3_(VO_4_)_2_‐0.5V_ZVO_. This is due to that acid etching also produces V and O vacancies and lead to the reduction of zinc valence state, confirming the existence of Zn─V─O vacancy clusters. The interatomic distances and coordination condition of Zn_3_(VO_4_)_2_ and Zn_3_(VO_4_)_2_‐0.5V_ZVO_ were determined from Fourier‐transformed (FT) EXAFS spectra of Zn K‐edge (Figure 4c). The peak at 1.5 Å corresponds to the Zn─O bond in the first coordination shell, and the peak strength of Zn_3_(VO_4_)_2_‐0.5V_ZVO_ is reduced compared with Zn_3_(VO_4_)_2_, indicating a lower coordination number of the Zn─O bond. In addition, the peak at 2.8 Å corresponds to the Zn─Zn bond, and its peak strength decreases sharply compared with Zn_3_(VO_4_)_2_. The coordination information proves the formation of unsaturated coordination oxygen atoms in Zn_3_(VO_4_)_2_‐0.5V_ZVO_. To visualize the coordination environments of Zn, wavelet transform (WT) plots of Zn_3_(VO_4_)_2_ and Zn_3_(VO_4_)_2_‐0.5V_ZVO_ are performed as shown in Figure 4d,e. The oscillations shown in the WT map correspond to the Zn─O bond and Zn─Zn bond, which reflect the coordination environment of Zn more directly, further confirming the existence of Zn─V─O vacancy clusters and unsaturated coordination oxygen atoms in Zn_3_(VO_4_)_2_‐0.5V_ZVO_.

Figure 4f shows the FT‐IR spectra of Zn_3_(VO_4_)_2_ and Zn_3_(VO_4_)_2_‐0.5V_ZVO_ in the wavelength region of 300–2000 cm^−1^. No water and hydroxyls vibration modes are detected owing to the high temperature annealing process. Appearance of peaks at 420 and 897 cm^−1^ is assigned to stretching vibration of V‐O‐Zn band [38]. The intense peaks at 629 and 827 cm^−1^ are due to V‐O‐V stretching in tetrahedral rocking vibration modes of VO_4_ [39]. A small sharp peak at 784 cm^−1^ is observed due to bonds shared by corner atoms of VO_4_ tetrahedral structure. By contrast, the overall chemical bond strength of Zn_3_(VO_4_)_2_‐0.5V_ZVO_ is lower than that of Zn_3_(VO_4_)_2_, indicating the existence of Zn─V─O vacancy clusters. To analyze the defect properties of Zn_3_(VO_4_)_2_‐V_ZVO_, electron paramagnetic resonance (EPR) test is performed (Figure 4g). Zn_3_(VO_4_)_2_ and Zn_3_(VO_4_)_2_‐0.5V_ZVO_ both show signals at g = 2.058. That is much stronger in Zn_3_(VO_4_)_2_‐0.5V_ZVO_ compared with Zn_3_(VO_4_)_2_, due to the presence of unpaired electrons in Zn_3_(VO_4_)_2_‐0.5V_ZVO_, further proving the formation of unsaturated coordination oxygen atoms. The compositions and chemical states of Zn_3_(VO_4_)_2_ and Zn_3_(VO_4_)_2_‐0.5V_ZVO_ are characterized by X‐ray photoelectron spectroscopy (XPS). The XPS full scan spectra confirm the presence of Zn, V, O and C elements in Zn_3_(VO_4_)_2_ and Zn_3_(VO_4_)_2_‐0.5V_ZVO_ (Figure S4). Figure S5a,b show the high‐resolution spectra of Zn 2p for Zn_3_(VO_4_)_2_ and Zn_3_(VO_4_)_2_‐0.5V_ZVO_, respectively. In Zn_3_(VO_4_)_2_‐0.5V_ZVO_, the characteristic peaks of Zn at 2p_3/2_ and 2p_1/2_ are located at 1021.2 and 1044.4 eV, respectively [40]. Compared with Zn_3_(VO_4_)_2_, the blueshift of Zn 2p in Zn_3_(VO_4_)_2_‐0.5V_ZVO_ is due to the accumulation of some negative oxygen ions near the Zn vacancy, resulting in increase in electron density around Zn and decrease of binding energy, further confirming the successfully introduce of unsaturated coordination oxygen atoms. As shown in Figure S5c,d, O 1s spectra of Zn_3_(VO_4_)_2_‐0.5V_ZVO_ can be fitted into two peaks, which are assigned to O^2−^ in Zn‐O and V‐O (529.6 eV) and O‐deficiency (531.0 eV), respectively [41, 42]. The characteristic peak of O‐deficiency in Zn_3_(VO_4_)_2_‐0.5V_ZVO_ is much stronger than that of Zn_3_(VO_4_)_2_, which indicates oxygen vacancies exist in Zn_3_(VO_4_)_2_‐0.5V_ZVO_. In the V2p spectra, each main peak can be fitted from two peaks derived from V^5+^ and V^4+^, respectively. In Zn_3_(VO_4_)_2_, the characteristic peaks of V^5+^ at 2P_3/2_ and 2P_1/2_ are at 516.8 and 524.3 eV, respectively. The two orbits of V^4+^ are at 515.9 and 522.3 eV, respectively [43]. Compared with Zn_3_(VO_4_)_2_, all the peaks of V2p in Zn_3_(VO_4_)_2_‐0.5V_ZVO_ shift in the direction of low binding energy (Figure S5e,f). Combined with the results of Zn 2p and O 1s, the formation of Zn─V─O vacancy clusters and unsaturated coordination oxygen atoms are further proved. Figure S6a,b respectively display the results of N_2_ adsorption/desorption isotherms and the pore size distributions of Zn_3_(VO_4_)_2_ and Zn_3_(VO_4_)_2_‐0.5V_ZVO_. Zn_3_(VO_4_)_2_ and Zn_3_(VO_4_)_2_‐0.5V_ZVO_ have a typical H4‐type loop, which merely arise from the presence of large mesopores. From the pore size distribution, Zn_3_(VO_4_)_2_ and Zn_3_(VO_4_)_2_‐0.5V_ZVO_ possess a relatively centralized pore size distribution below 60 nm. It is worth noting that in Zn_3_(VO_4_)_2_‐0.5V_ZVO_, the acid etch increases the specific surface area from 6.98 to 132.29 m^2^ g^−1^ and pore volume from 0.05 to 0.30 cm^3^ g^−1^. This will increase the contact area between the electrode and electrolyte, improving its electrochemical performance. Thermogravimetric test shows that Zn_3_(VO_4_)_2_ and Zn_3_(VO_4_)_2_‐0.5V_ZVO_ both have little carbon content, which are 0.2% and 0.4%, respectively (Figure S6c). As shown in Figure 4h, contact angles of Zn_3_(VO_4_)_2_ and Zn_3_(VO_4_)_2_‐0.5V_ZVO_ are 22.87° and 9.55°, respectively, indicating an enhanced hydrophilicity of Zn_3_(VO_4_)_2_‐0.5V_ZVO_. That will fully increase the contact area between the electrode material and electrolyte.

**FIGURE 5:**
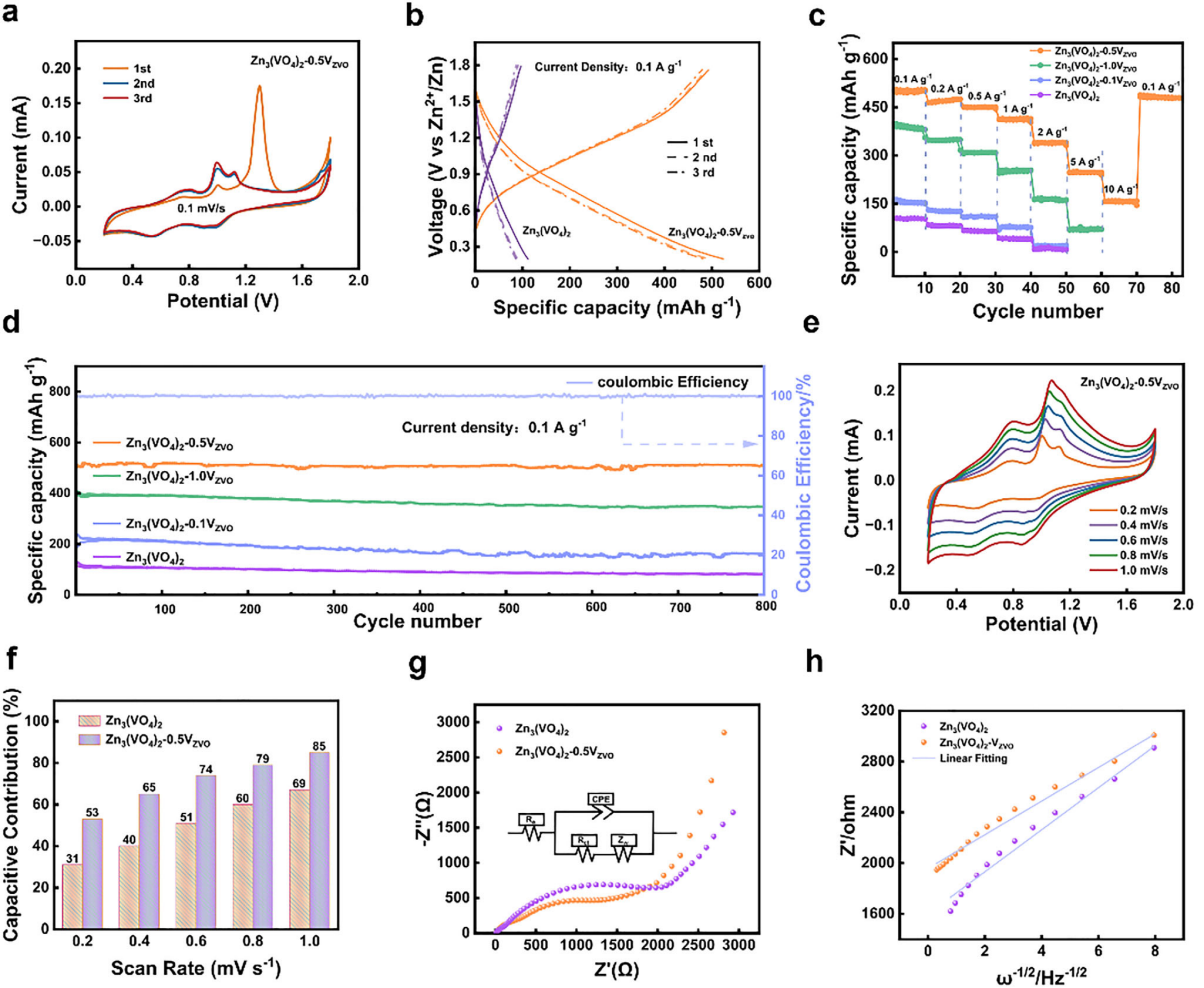
(a) CV curves of Zn_3_(VO_4_)_2_‐0.5V_ZVO_ cathode at 0.1 mV s^−1^; (b) The galvanostatic charge/discharge profiles of Zn_3_(VO_4_)_2_ and Zn_3_(VO_4_)_2_‐0.5V_ZVO_ cathode at 0.1 A g^−1^; (c) Rate performances and (d) cyclic performances of Zn_3_(VO_4_)_2_, Zn_3_(VO_4_)_2_‐0.1V_ZVO_, Zn_3_(VO_4_)_2_‐0.5V_ZVO_ and Zn_3_(VO_4_)_2_‐1.0V_ZVO_ cathodes; (e) CV curves of Zn_3_(VO_4_)_2_‐0.5V_ZVO_ at different scan rates from 0.2 to 1.0 mV s^−1^; (f) The calculated capacitive contribution rates in Zn_3_(VO_4_)_2_ and Zn_3_(VO_4_)_2_‐0.5V_ZVO_ cathode at different scan rates; (g) EIS curves of Zn_3_(VO_4_)_2_ and Zn_3_(VO_4_)_2_‐0.5V_ZVO_ cathodes; (h) Warburg factors of Zn_3_(VO_4_)_2_ and Zn_3_(VO_4_)_2_‐0.5V_ZVO_ cathodes.

Electrochemical tests of Zn_3_(VO_4_)_2_ and Zn_3_(VO_4_)_2_‐V_ZVO_ cathode are conducted to investigate the effect of Zn─V─O vacancy clusters on the zinc storage performance. Cyclic voltammetry (CV) tests are performed on Zn_3_(VO_4_)_2_ and Zn_3_(VO_4_)_2_‐0.5V_ZVO_ electrodes at a scan rate of 0.1 mV s^−1^ and a potential window of 0.2‐1.8 V (Figure 5a; Figure S7). The first CV scanning cycle of Zn_3_(VO_4_)_2_‐0.5V_ZVO_ is slightly different from the subsequent cycles due to the electrochemical activation of the electrode material during the initial cycle [44]. In the following cycles, the two redox peaks are assigned to the electrochemical removal of H_2_O and Zn^2+^ [45]. It can be seen that the redox sites of Zn_3_(VO_4_)_2_ do not make a difference in the first three cycles of CV curve. The charge/discharge profiles of Zn_3_(VO_4_)_2_‐0.5V_ZVO_ exhibit two typical plateaus at 0.9‐1.1 V and 1.0 V (Figure 5b), as fully conformed with the CV curves. Compared with Zn_3_(VO_4_)_2_, the charging and discharging platform is more obvious in Zn_3_(VO_4_)_2_‐0.5V_ZVO_, indicative of the more expedite zinc ion diffusion paths derived from the abundant Zn─V─O vacancy clusters. The rate properties of Zn_3_(VO_4_)_2_, Zn_3_(VO_4_)_2_‐0.5V_ZVO_ and the other etched samples (Zn_3_(VO_4_)_2_‐0.1V_ZVO_, Zn_3_(VO_4_)_2_‐1.0V_ZVO_) at different current densities are shown in Figure 5c and Figure S8. The reversible capacities of Zn_3_(VO_4_)_2_‐0.5V_ZVO_ are 517, 469, 445, 410, 338, 247, 156 and 489 mAh g^−1^, respectively, when the current densities increase from 0.1 to 0.2, 0.5, 1, 2, 5 and 10 A g^−1^, and then decrease to 0.1 A g^−1^.

However, the reversible capacities of Zn_3_(VO_4_)_2_ are only 102, 84, 81, 59, 47 mAh g^−1^ at the corresponding current densities, and it can barely sustain effective cycling at current densities of 5 A g^−1^ and above, with its specific capacity rapidly decaying to nearly zero. These values are much lower than those of the Zn_3_(VO_4_)_2_‐V_ZVO_ series samples, indicating that the vacancy clusters provide more active sites for electron and ion transfer and facilitate the deintercalation of zinc ions. Notably, the rate capability of Zn_3_(VO_4_)_2_‐0.5V_ZVO_ is superior to that of most reported vanadium‐based cathode materials for AZIBs (Figure S9 and Table S2) [46, 47, 48, 49, 50, 51, 52]. As shown in Figure 5d, after 800 cycles at 0.1 A g^−1^, the specific capacities of Zn_3_(VO_4_)_2_‐0.5V_ZVO_ and Zn_3_(VO_4_)_2_ are 506 and 83 mAh g^−1^, respectively, indicating that the vacancies clusters is not only conducive to inhibiting the volume expansion during Zn^2+^ deintercalation, but also conducive to promoting Zn^2+^ deintercalation determined by the unsaturated coordination oxygen atoms. The initial coulombic efficiencies of Zn_3_(VO_4_)_2_‐0.5V_ZVO_ and Zn_3_(VO_4_)_2_ electrodes are 93.9% and 86.5%, respectively. In the subsequent charge‐discharge cycles, coulombic efficiency of Zn_3_(VO_4_)_2_‐0.5V_ZVO_ is close to 100%.

Meanwhile, it can maintain excellent capacity retention over 400 cycles at 5 A g^−1^, and its performance is significantly superior to that of the pristine Zn_3_(VO_4_)_2_ as well as the two doped samples Zn_3_(VO_4_)_2_‐1.0V_ZVO_ and Zn_3_(VO_4_)_2_‐0.1V_ZVO_ (Figure S10). These results confirm that the Zn_3_(VO_4_)_2_‐0.5V_ZVO_ electrode exhibits outstanding cycling stability not only at a low current density (0.1 A g^−1^) but also at a high current density (5 A g^−1^), demonstrating its great potential for high‐power energy storage applications. In addition, we have assessed the electrochemical performance at elevated active material loadings and have supplemented the cycling data for the Zn_3_(VO_4_)_2_‐0.5V_ZVO_ electrode with an active material loading of 2.0 mg cm^−2^ in Figure S11. At a current density of 0.1 A g^−1^, this electrode delivers a stable specific capacity of ∼320 mAh g^−1^. The prolonged electron diffusion pathways and increased internal resistance under high‐loading conditions result in a minor reduction in specific capacity with increasing loading. Despite this, the electrode maintains excellent cycling stability, verifying the promising potential of this material for practical energy storage applications even at higher active material loadings.

To research the storage behavior of Zn^2+^ in Zn_3_(VO_4_)_2_ and Zn_3_(VO_4_)_2_‐0.5V_ZVO_, the scanning voltammetry curves of Zn_3_(VO_4_)_2_ and Zn_3_(VO_4_)_2_‐0.5V_ZVO_ at different scanning rates ranging from 0.2 to 1.0 mV s^−1^ are performed in Figure 5e and Figure S12. The electrode peak area of Zn_3_(VO_4_)_2_‐0.5V_ZVO_ is much wider than that of Zn_3_(VO_4_)_2_, which indicates that Zn^2+^ is better adsorbed on Zn_3_(VO_4_)_2_‐0.5V_ZVO_ electrode, and it will have a greater capacitance contribution.

The *b*‐values of oxidation and reduction peaks for Zn_3_(VO_4_)_2_‐0.5V_ZVO_ are 0.88 and 0.81, respectively (Figure S13a). The calculated *b*‐values of reduction and oxidation peaks for Zn_3_(VO_4_)_2_ are 0.69 and 0.72, respectively (Figure S13b). It indicates that the energy storage process in Zn_3_(VO_4_)_2_ and Zn_3_(VO_4_)_2_‐0.5V_ZVO_ is jointly controlled by both diffusion behavior and capacitive behavior, with capacitive behavior playing a dominant role [53]. Figure S14 represents the calculated pseudocapacitance contribution rates of Zn_3_(VO_4_)_2_‐0.5V_ZVO_ and Zn_3_(VO_4_)_2_ at a scanning rate of 1.0 mV s^−1^, which are 85% and 67%, respectively. As the scanning rate increasing, the capacitive contributions of Zn_3_(VO_4_)_2_‐0.5V_ZVO_ and Zn_3_(VO_4_)_2_ demonstrate an increasing trend (Figure 5f; Figures S15 and S16).

To further investigate the electrochemical kinetics of the electrodes, the electrochemical impedance spectroscopy (EIS) test is performed (Figure 5g). The high frequency intercept at Z′ axis is the combined resistance of the electrolyte and cell components (*R_e_*). The high‐middle frequency semicircle is the charge‐transfer resistance (*R_ct_*) at the interface between the electrolyte and electrode. The low‐frequency oblique line represents the Warburg impedance (W), which belongs to Zn^2+^ ion diffusion process in the electrode. The lower *R_ct_* was found for Zn_3_(VO_4_)_2_‐0.5V_ZVO_ electrode, indicating the lower resistance to charge transfer and faster Zn‐intercalation kinetics. The Zn─V─O vacancy clusters in Zn_3_(VO_4_)_2_‐0.5V_ZVO_ could significantly increase the conductivity, and the introduced unsaturated coordination oxygen atoms makes the insertion and extraction of ions easier. The diffusion coefficients of Zn^2+^ (D_Zn2+_) were calculated according to the measured impedance fitting curve (Figure 5h) [54]. The calculated D_Zn2+_ of Zn_3_(VO_4_)_2_ and Zn_3_(VO_4_)_2_‐0.5V_ZVO_ are 5.43 × 10^−19^, 8.85 × 10^−19^ cm^2^/s, respectively. Zn─V─O vacancy clusters in Zn_3_(VO_4_)_2_‐0.5V_ZVO_ make a large amount of free space, allowing the electrolyte to fully permeate and shorten the diffusion path of Zn^2+^.

**FIGURE 6:**
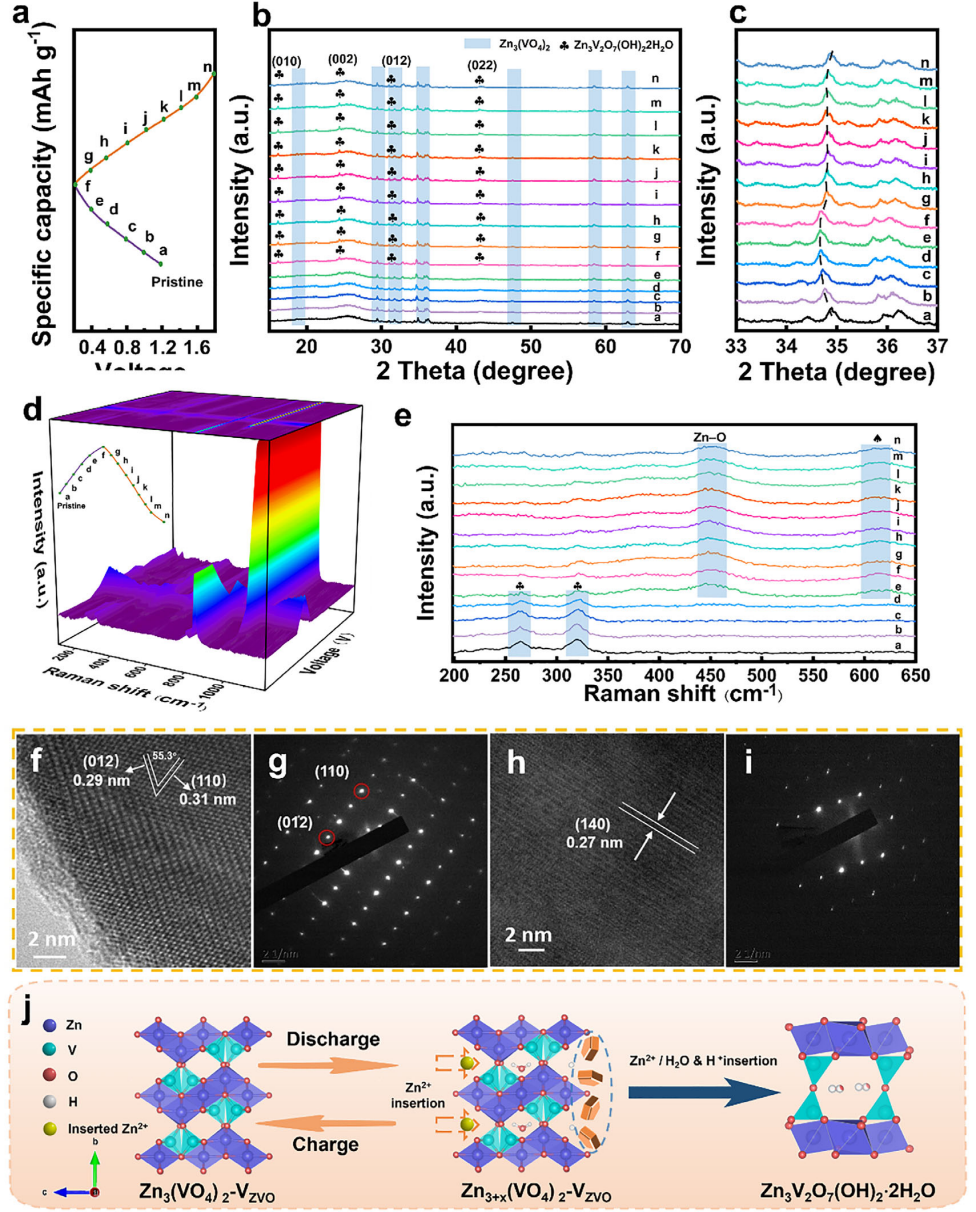
(a‐c) *In‐*s*itu* XRD patterns in the first cycle of Zn_3_(VO_4_)_2_‐0.5V_ZVO_; (d‐e) *In*‐situ Raman spectra of Zn_3_(VO_4_)_2_‐0.5V_ZVO_ cathode collected at the selected states at the first cycle; (f) HRTEM image and (g) SAED spectra of Zn_3_V_2_O_7_(OH)_2_·2H_2_O; (h) HRTEM image and (i) SAED spectra of Zn_3_(VO_4_)_2_‐0.5V_ZVO_ after cycling; (j) Zinc storage mechanism of Zn_3_(VO_4_)_2_‐Vzvo cathode.

To verify the zinc ion storage mechanism of the Zn_3_(VO_4_)_2_‐0.5V_ZVO_ cathode, we performed *in‐situ* XRD, *in‐situ* Raman and HRTEM after discharging. In Figure 6a,b,c and Figure S17, it is worth noting that new peaks appear at 16.2°, 24.3°, 30.1°, and 42.7° when discharging from 1.2‐0.2 V, which are attributed to (110), (002), (012) and (022) plane of Zn_3_V_2_O_7_(OH)_2_·2H_2_O (PDF#00‐050‐0570) [55]. Subsequently, when charging from 0.2 to 1. 8 V (f‐n), the four peaks still exist. This indicates that Zn_3_V_2_O_7_(OH)_2_·2H_2_O is gradually formed with the insertion of zinc ions. In Figure 6c, the 2*θ* value of (210) peak shifts from 34.88° to 34.64° when discharging from 1.2 to 0.2 V (a‐f). This indicates that Zn^2+^ and H^+^ are inserted into Zn_3_(VO_4_)_2_‐0.5V_ZVO_ layers, causing an expansion of the interlayer spacing. When charging back to 1.8 V, the (210) peak returns to 34. 88°, ascribing to the desertion of Zn^2+^ and H^+^ from Zn_3_(VO_4_)_2_‐0.5V_ZVO_. In Figure 6d,e, during the discharge of Zn_3_(VO_4_)_2_‐0.5V_ZVO_, the peak intensity at 850 cm^−^^1^ (V─O single bond) decreases, while that at 980 cm^−^^1^ (V═O double bond) increases, indicating the transformation of V─O single bonds into double bonds [56, 57]. This reflects the structural transition from tetrahedral coordination to bridged dimers, accompanied by reconstruction of coordination configurations. The dissociation of VO_4_ tetrahedra and their reorganization into V_2_O_7_ dimers, along with embedding of hydroxyl groups and water molecules, leads to a change in the proportion of V─O bond types: the proportion of bridged single bonds decreases, whereas that of terminal double bonds increases. The attenuation of peak intensities at 264 and 319 cm^−^^1^ (coupled vibrations between VO_4_ tetrahedra) indicates cleavage of long‐range V─O─V bridging bonds shared by tetrahedra and their transformation into intra‐dimer linkages. The newly emerging stretching vibration peak of Zn─O bonds at 450 cm^−^^1^ confirms that embedding of Zn^2+^ forms a new phase containing Zn─O bonds [58]. The peak at 620 cm^−^^1^ originates from the coupled vibration of V─O and Zn─O bonds, resulting from softening of V─O bonds and reconstruction of vibration modes induced by Zn^2+^ insertion. Collectively, these phenomena reflect crystal structure transition and metal‐oxygen framework modifications induced by Zn^2+^ insertion, along with alteration of vibration energy transfer pathways. By contrast, the unetched sample does not exhibit the above‐mentioned phenomena due to its robust tetrahedral coordination structure. The formation of Zn_3_V_2_O_7_(OH)_2_·2H_2_O after discharging is also verified by HRTEM in Figure 6f,g. When discharged to 0.2 V, the *d*‐spacings are found to be 0.29 and 0.31 nm, corresponding to (012) and (110) plane of Zn_3_V_2_O_7_(OH)_2_·2H_2_O, respectively. In addition, (140) plane of Zn_3_(VO_4_)_2_‐0.5V_ZVO_ increases to 0.27 nm due to the intercalation of Zn^2+^ (Figure 6h,i). It is worth noting that the *ex ‐situ* XRD pattern of the second and third cycles also indicate the generating of Zn_3_V_2_O_7_(OH)_2_·2H_2_O (FigureS S18 and S19). That indicates that Zn_3_V_2_O_7_(OH)_2_·2H_2_O is cyclic involved in the deintercalation process of zinc ions during charge and discharge process. As shown in Figure S20, the *ex‐situ* XRD patterns of Zn_3_(VO_4_)_2_ reveal the same reaction process as Zn_3_(VO_4_)_2_‐0.5V_ZVO_.

According to the above analysis combined with the initial AIMD results, the summary of the charge/discharge processes on the electrode is as follows:

(1) The reaction on the zinc anode: $\text{Zn}\underset{\text{Charge}}{\overset{\text{Discharge}}{⇄}\;}Zn^{2+}+2e^{-}$
(2) The reactions on cathode:
  (a) $Zn_{3}{\left(VO_{4}\right)}_{2}-V_{\text{ZVO}}+xZn^{2+}+H^{+}+H_{2}O\underset{\text{Charge}}{\overset{\text{Discharge}}{⇄}\;}\;Zn_{3+x}V_{2}O_{7}{\left(\text{OH}\right)}_{2}\cdot 2H_{2}O$
  (b) The adsorption of Zn^2+^ and H^+^ in Zn_3_(VO_4_)_2_‐V_ZVO_

The proposed multi‐ion/molecule intercalation/extraction process occurring in Zn_3_(VO_4_)_2_‐V_ZVO_ during a typical cycle is summarized in Figure 6j. In reaction (b), Zn_3_(VO_4_)_2_‐V_ZVO_ does not experience any phase/structure transformation; the only change is its gallery spacing to accommodate Zn^2+^, H^+^. Combined with the initial AIMD analysis, the reaction (a) on cathode contributes partial capacity and reaction (b) contributes the main capacity. It is worth emphasizing that in Zn_3_(VO_4_)_2_‐0.5V_ZVO_, the vacancy clusters greatly improve the adsorption of zinc ions in reaction (a) and (b), deriving from the abundant unsaturated coordination oxygen atoms compared with Zn_3_(VO_4_)_2_. The above processes are reversed during the charge cycle.

## 4 Conclusion

In summary, the unsaturated coordination oxygen atoms dominated Zn_3_(VO_4_)_2_‐V_ZVO_ has been synthesized and successfully used as the cathode material for AZIBs. Derived from the vacancy clusters, abundant unsaturated coordination oxygen atoms effectively capture most free Zn^2^^+^ and H^+^ ions, reduce inherent electrostatic interactions, and contribute dominative capacity. Meanwhile, part of the adsorbed Zn^2^^+^ can reversibly intercalate Zn_3_(VO_4_)_2_‐V_ZVO_ and transform into Zn_3_V_2_O_7_(OH)_2_·2H_2_O to contribute the remaining capacity. This study reveals the formation and action mechanism of unsaturated coordination oxygen sites in Zn_3_(VO_4_)_2_‐V_ZVO_, and the novel strategy for designing such materials offers new insight into the development of high‐performance cathode materials for AZIBs.

## Conflicts of Interest

The authors declare no conflicts of interest.

## Supporting information




**Supporting File**: advs74996‐sup‐0001‐SuppMat.docx.

## Data Availability

The data that support the findings of this study are available from the corresponding author upon reasonable request.
