# Supplementary material for: Unsaturated Coordination Oxygen in Zn─V─O Vacancy Clusters Enables Superb Zinc Storage Capability
Source: Adv Sci (Weinh). 2026 Mar 26;13(32):e74996. doi: 10.1002/advs.74996 (PMC13252607; doi:10.1002/advs.74996)
Supplement: Supplementary file 1 — Supporting File: advs74996‐sup‐0001‐SuppMat.docx. [file ADVS-13-e74996-s001.docx]

*Supporting Online Materials for*

**Unsaturated Coordination Oxygen in Zn-V-O Vacancy Clusters Enables Superb Zinc Storage Capability**

Yulong Chi ^a, b, #^, Fulong Li ^c, #^, Yangxian Wang ^a, b, #^, Longwei Li ^a^, Haolin Li ^a^, Xiaodong Shi ^c^, Xinlong Tian ^c,^ *, Yihui Zou ^a, b,^ *, Dongjiang Yang ^a, b,^ *

^a^ Institute of Micro/Nano Materials and Devices, Ningbo University of Technology, Ningbo 315211, Zhejiang, P. R. China.

^b^ School of Environment and Geography, State Key Laboratory of Bio-fibers and Eco-textiles, Shandong Collaborative Innovation Center of Marine Biobased Fibers and Ecological Textiles, Institute of Marine Biobased Materials, Qingdao University, Qingdao 266071, P. R. China.

^c^ School of Marine Science and Engineering, State Key Laboratory of Tropic Ocean Engineering Materials and Materials Evaluation, Hainan University, Haikou 570228, P. R. China.

^#^ These authors contributed equally to this work.

**1. Chemicals and materials**

Ammonium metavanadate (NH_4_VO_3_), Carboxylated chitosan ((C_6_H_11_NO_4_)_n_)*,* Sodium alginate ((C_6_H_7_NaO_6_)_n_), Zinc acetate (Zn(CH_3_COO)_2_), Zinc sulfate (ZnSO_4_) and Hydrochloric acid (HCl) were purchased from Sinopharm reagent Group Co., Ltd.

**Supplementary figures and captions**


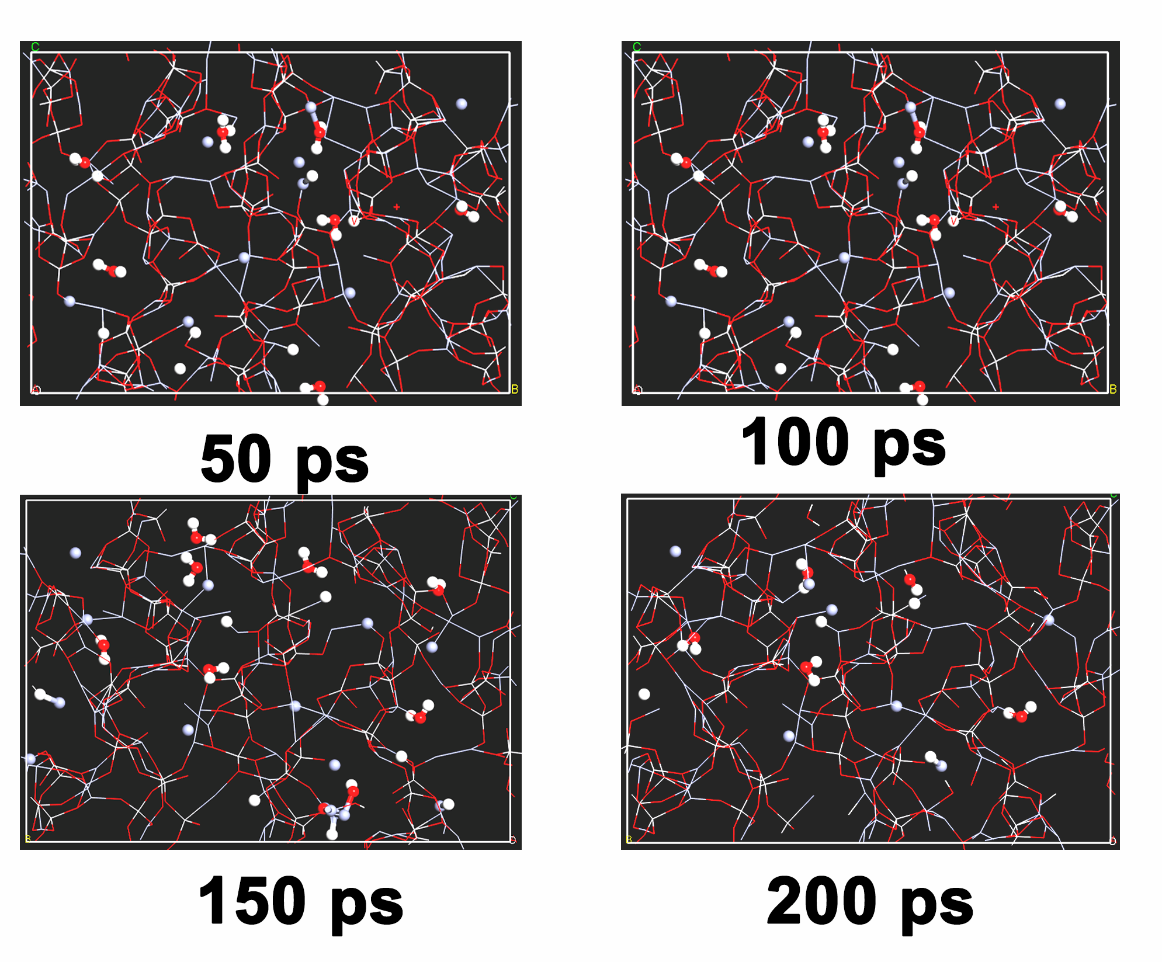


**Figure. S1** Snapshots of typical adsorption process for Zn^2+^, H^+^, H_2_O in Zn_3_(VO_4_)_2_.


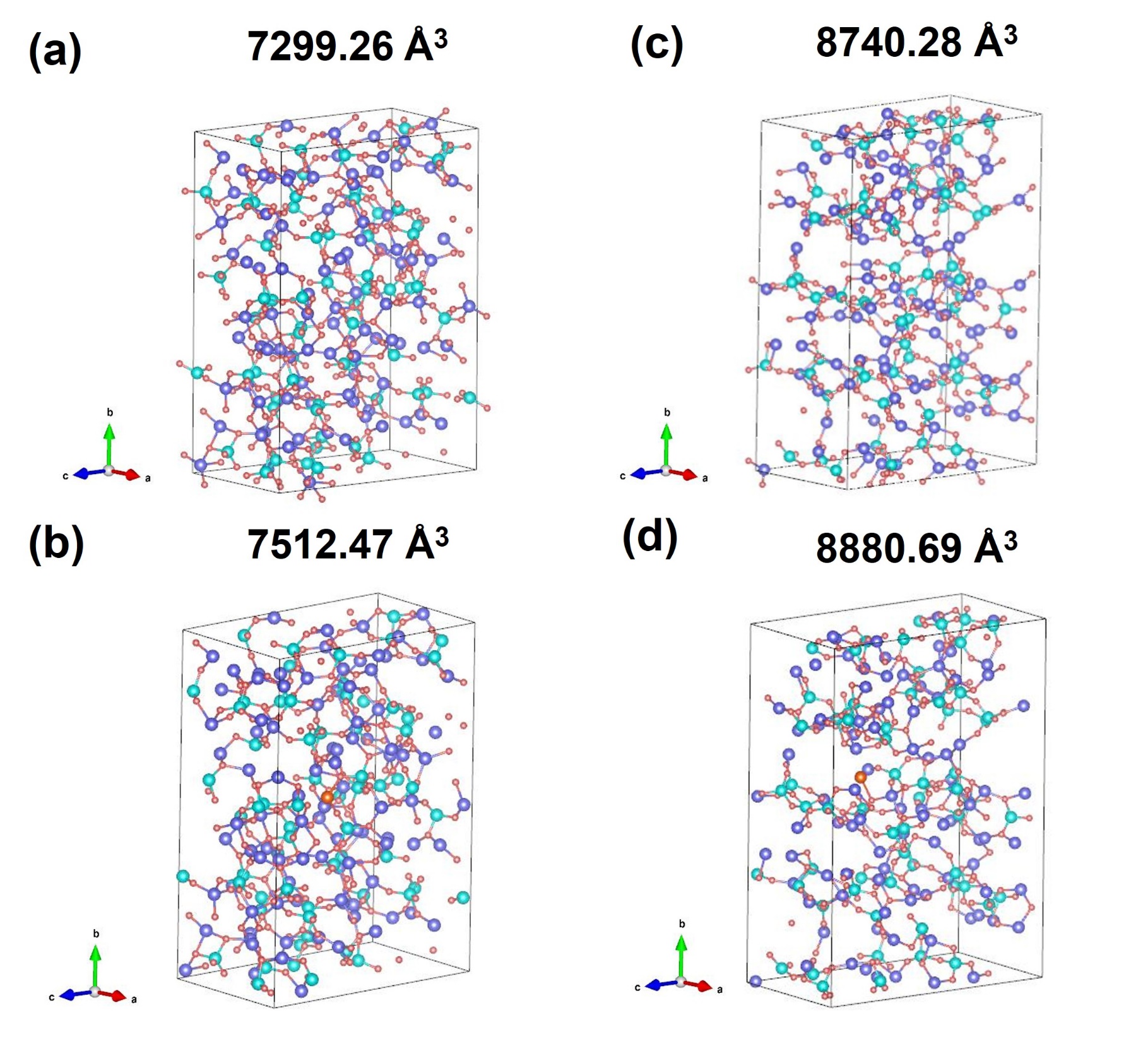


**Figure. S2** Volume change (a) before and (b) after Zn^2+^ adsorption in the Zn_3_(VO_4_)_2_; Volume change (c) before and (d) after Zn^2+^ adsorption in the Zn_3_(VO_4_)_2_-V_ZVO_.

**
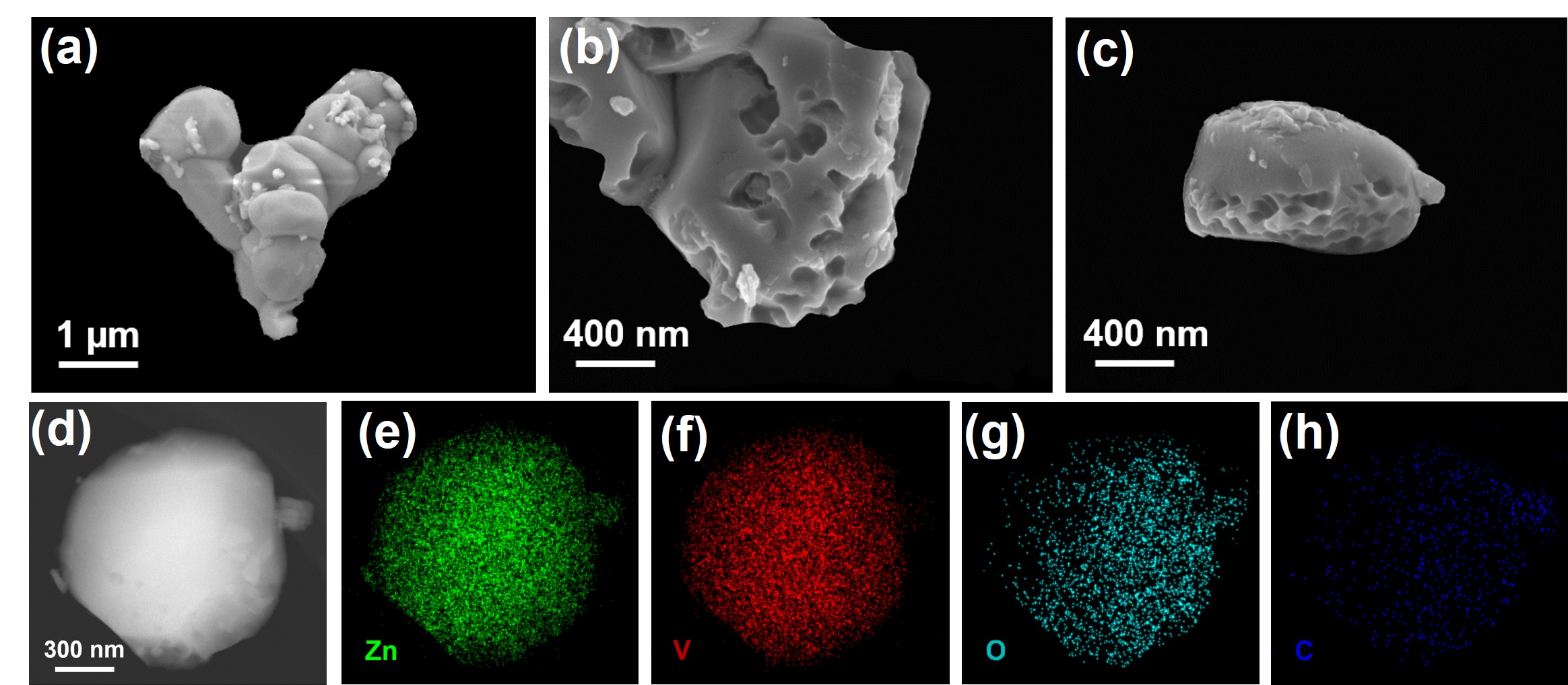
**

**Figure. S3** SEM images of (a) Zn_3_(VO_4_), and (b,c) Zn_3_(VO_4_)_2_-0.5V_ZVO_; (d) High angle annular dark field (HAADF) image of Zn_3_(VO_4_)_2_-0.5V_ZVO_ and the corresponding EDS mapping images for (e) Zn, (f) V, (g) O, and (h) C elements.


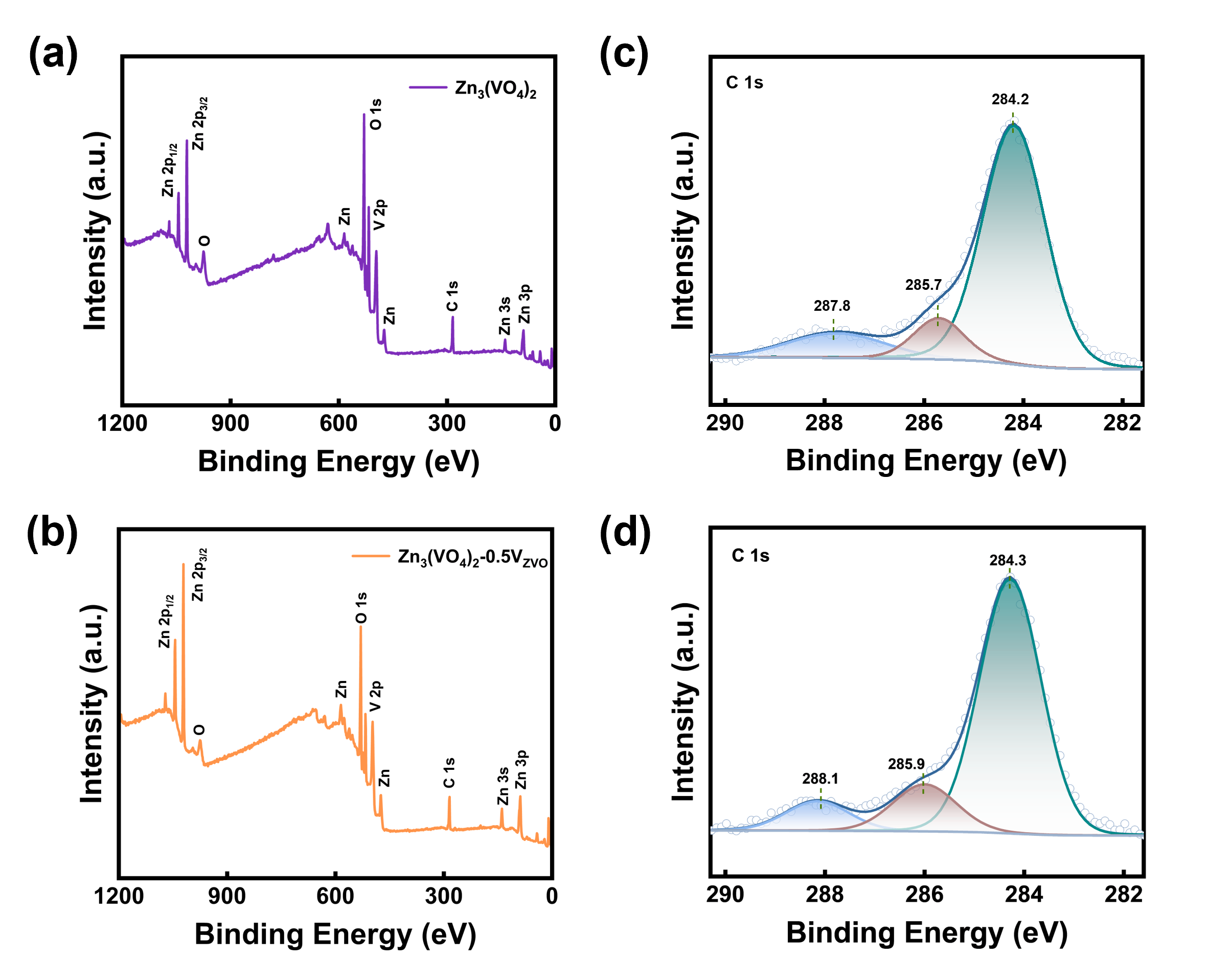


**Figure. S4** XPS survey spectra of (a) Zn_3_(VO_4_) and (b) Zn_3_(VO_4_)_2_-0.5V_ZVO_; High-resolution C 1s spectra of (c) Zn_3_(VO_4_) and (d) Zn_3_(VO_4_)_2_-0.5V_ZVO_.


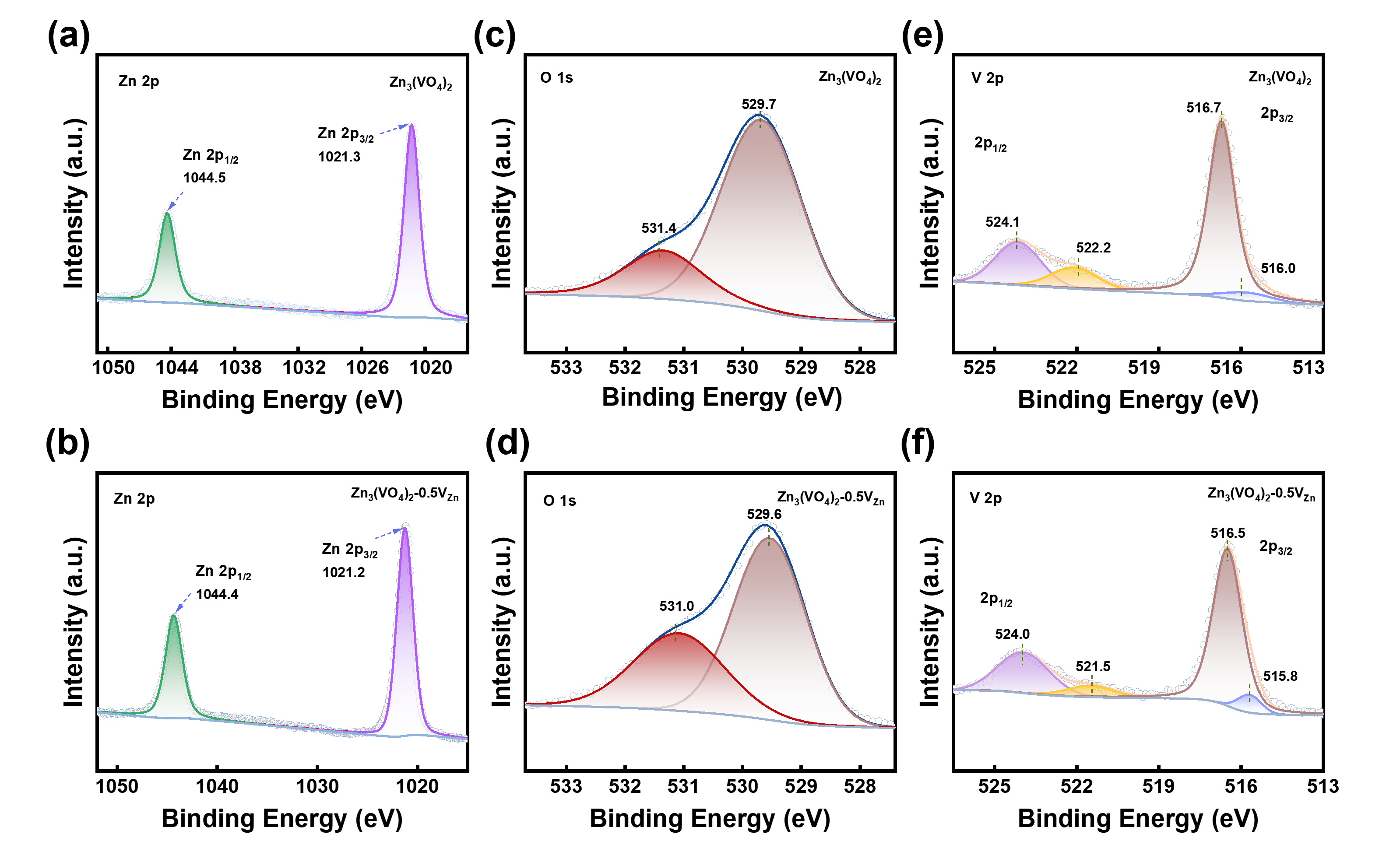


**Figure. S5** High-resolution (a) Zn 2p, (c) O 1s and (e) V 2p spectra of Zn_3_(VO_4_)_2_; High-resolution (b) Zn 2p, (d) O 1s and (f) V 2p spectra of Zn_3_(VO_4_)_2_-0.5V_ZVO_.


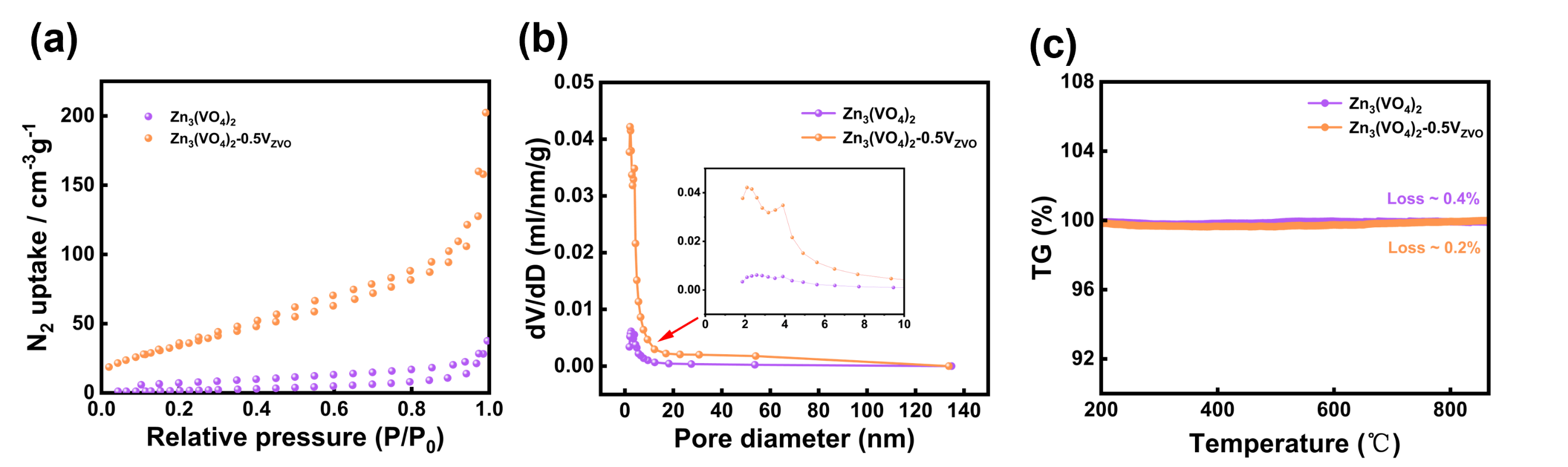


**Figure. S6** (a) N_2_ isothermal adsorption/desorption curves, (b) Pore size distribution profiles, and (c) thermogravimetric test curve of Zn_3_(VO_4_)_2_ and Zn_3_(VO_4_)_2_-0.5V_ZVO_.


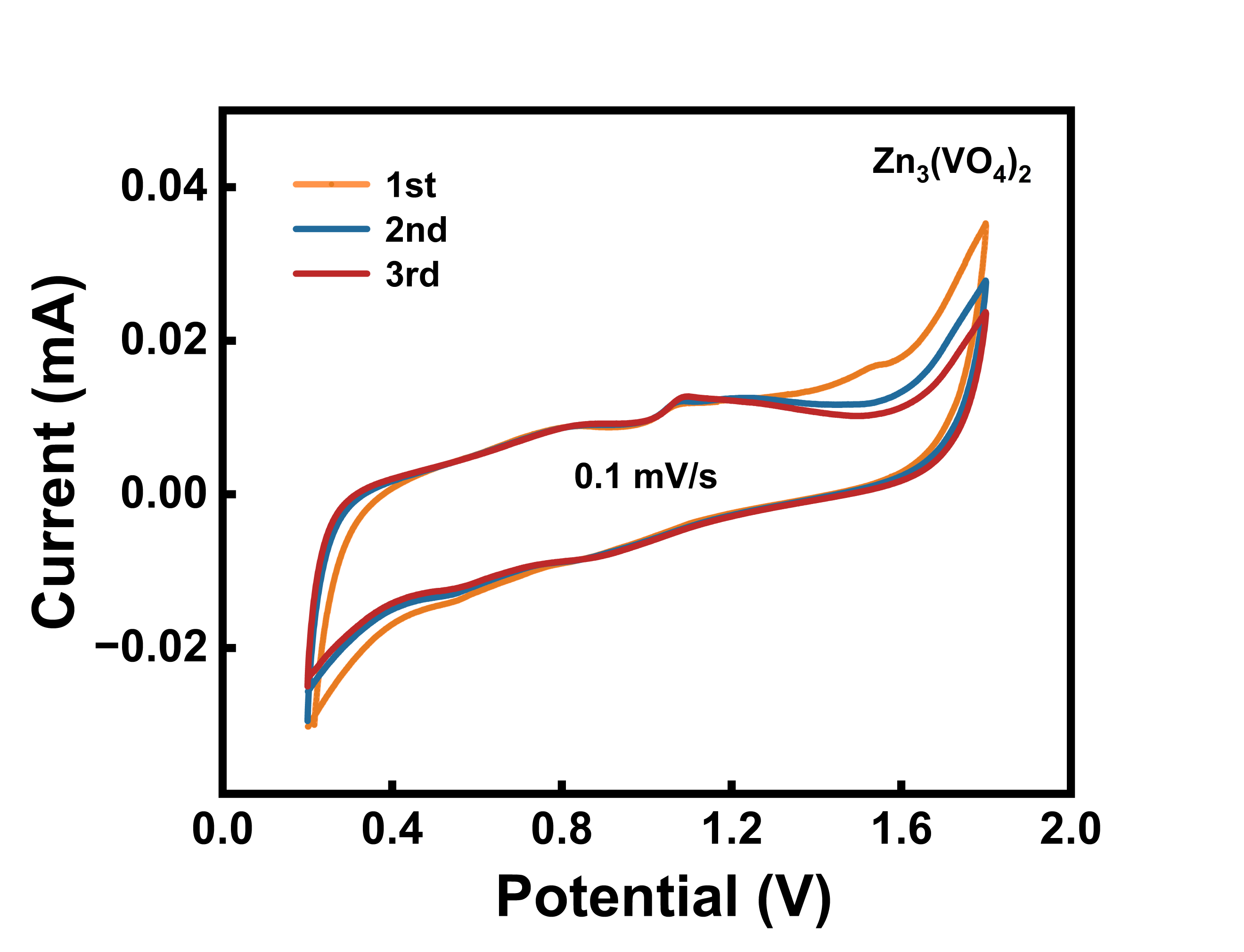


**Figure. S7** CV curves of Zn_3_(VO_4_) cathode at 0.1 mV s^-1^.

.


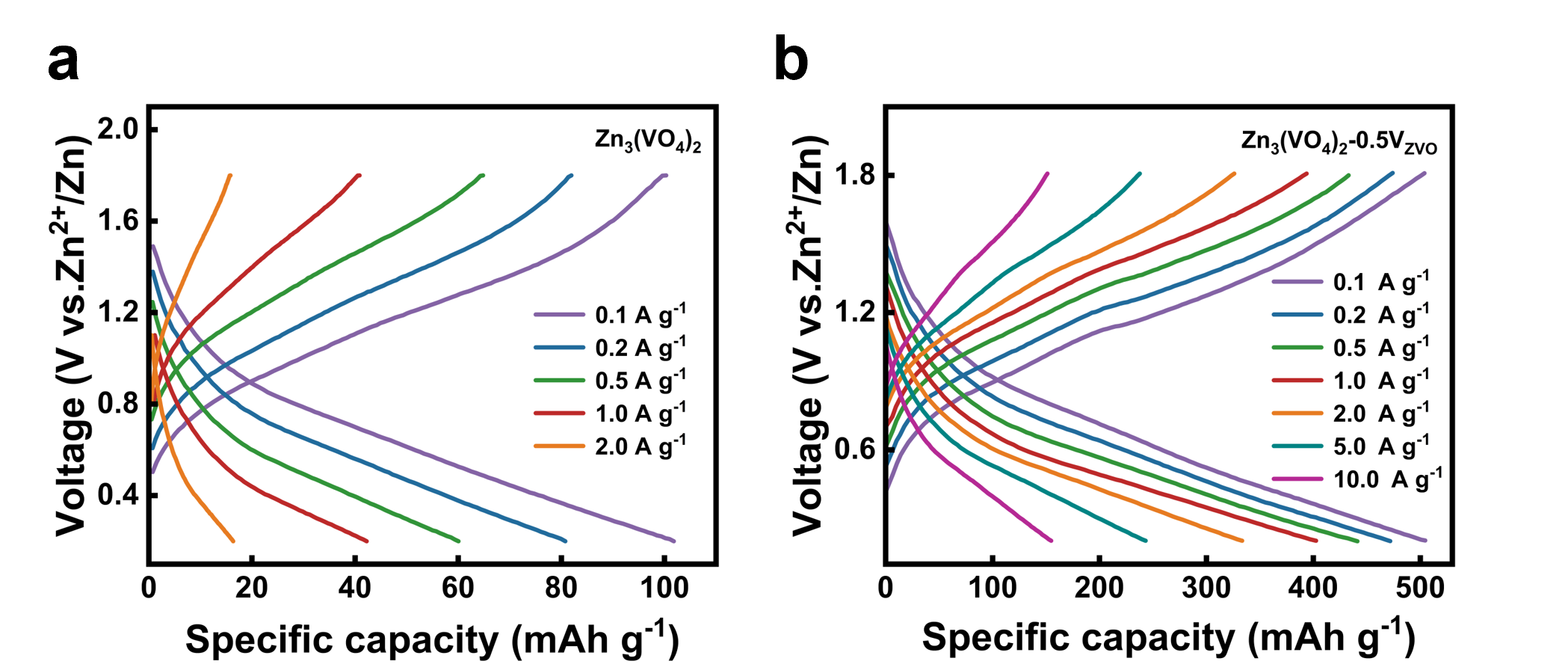


**Figure. S8** Galvanostatic charge/discharge profiles of (a) Zn_3_(VO_4_)_2_ and (b) Zn_3_(VO_4_)_2_-0.5V_ZVO_ cathode at diffident current densities.


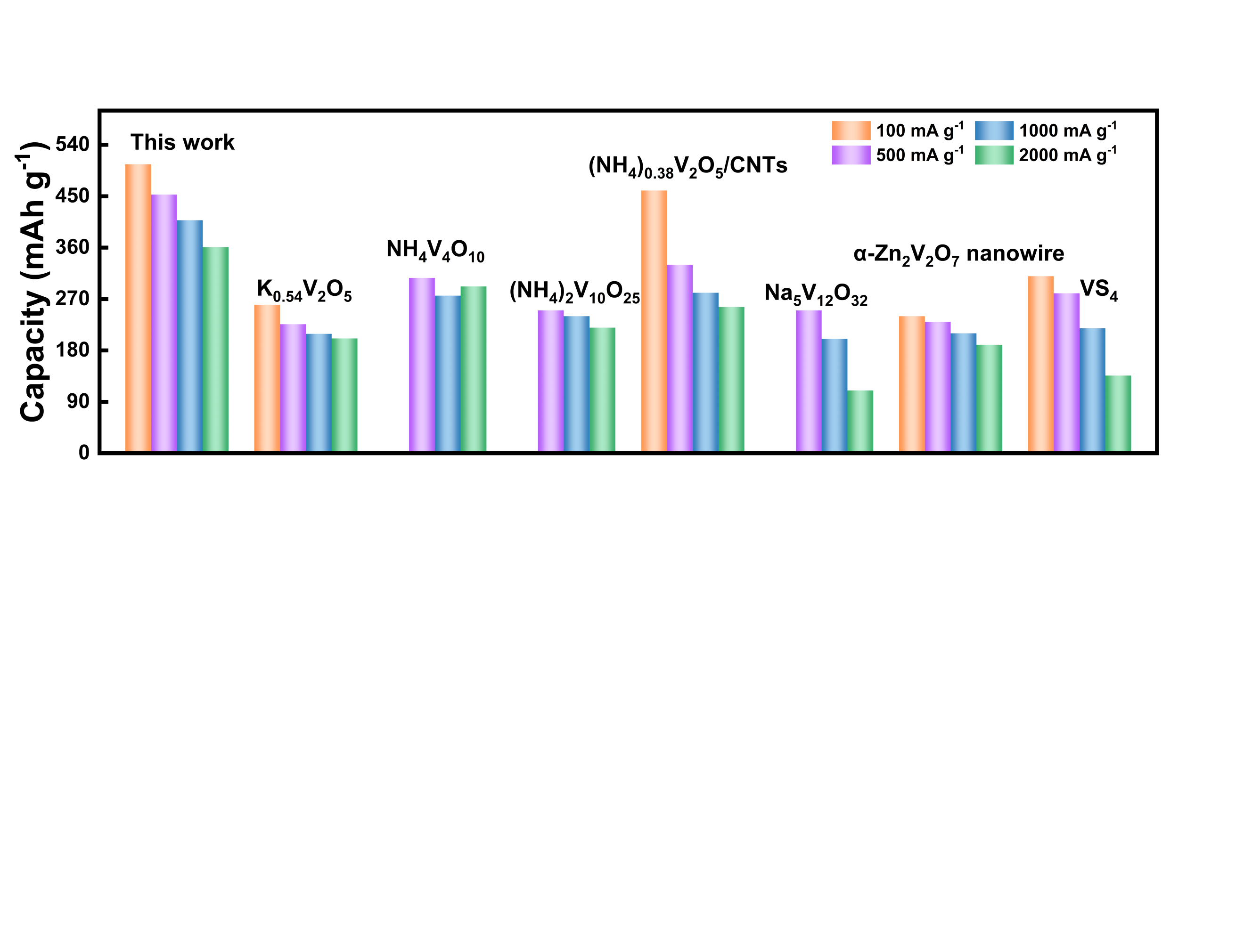


**Figure. S9** Comparison of zinc storage performances of Zn_3_(VO_4_)_2_-0.5V_ZVO_ with previously reported vanadates cathodes.


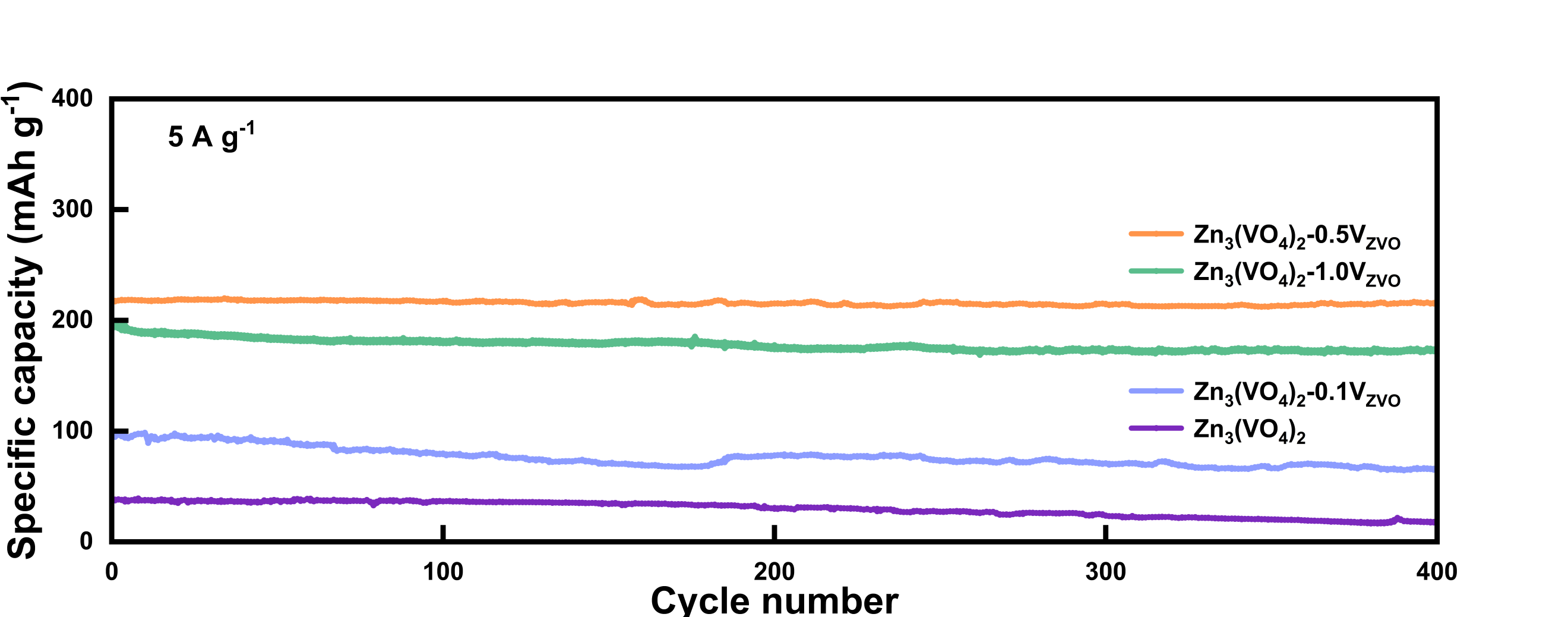


**Figure. S10** Cycling performances of Zn_3_(VO_4_)_2_, Zn_3_(VO_4_)_2_-0.1V_ZVO_, Zn_3_(VO_4_)_2_-0.5V_ZVO_, and Zn_3_(VO_4_)_2_-1.0V_ZVO_ cathodes at a current density of 5 A g^-1^.


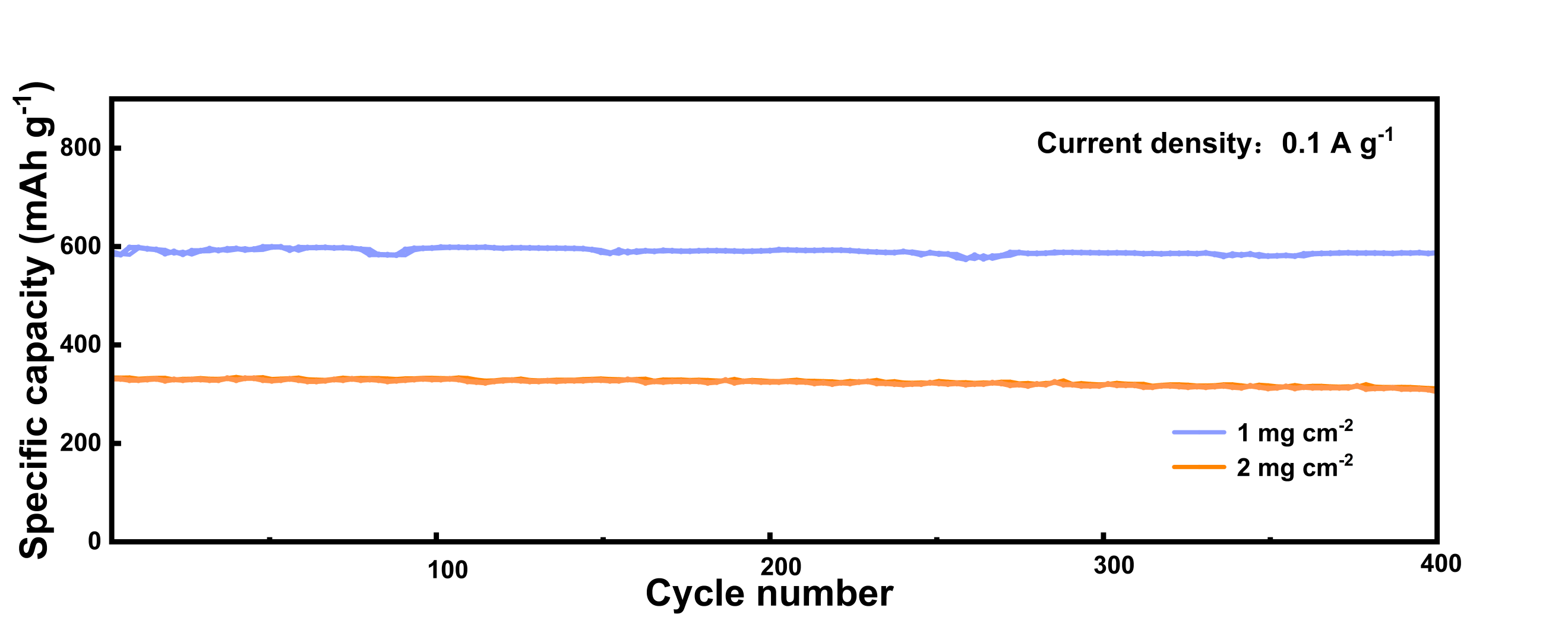


**Figure. S11** Cycling performance of Zn_3_(VO_4_)_2_-0.5V_ZVO_ electrodes with different mass loadings (1.0 and 2.0 mg cm^-2^) at a current density of 0.1 A g^-1^.


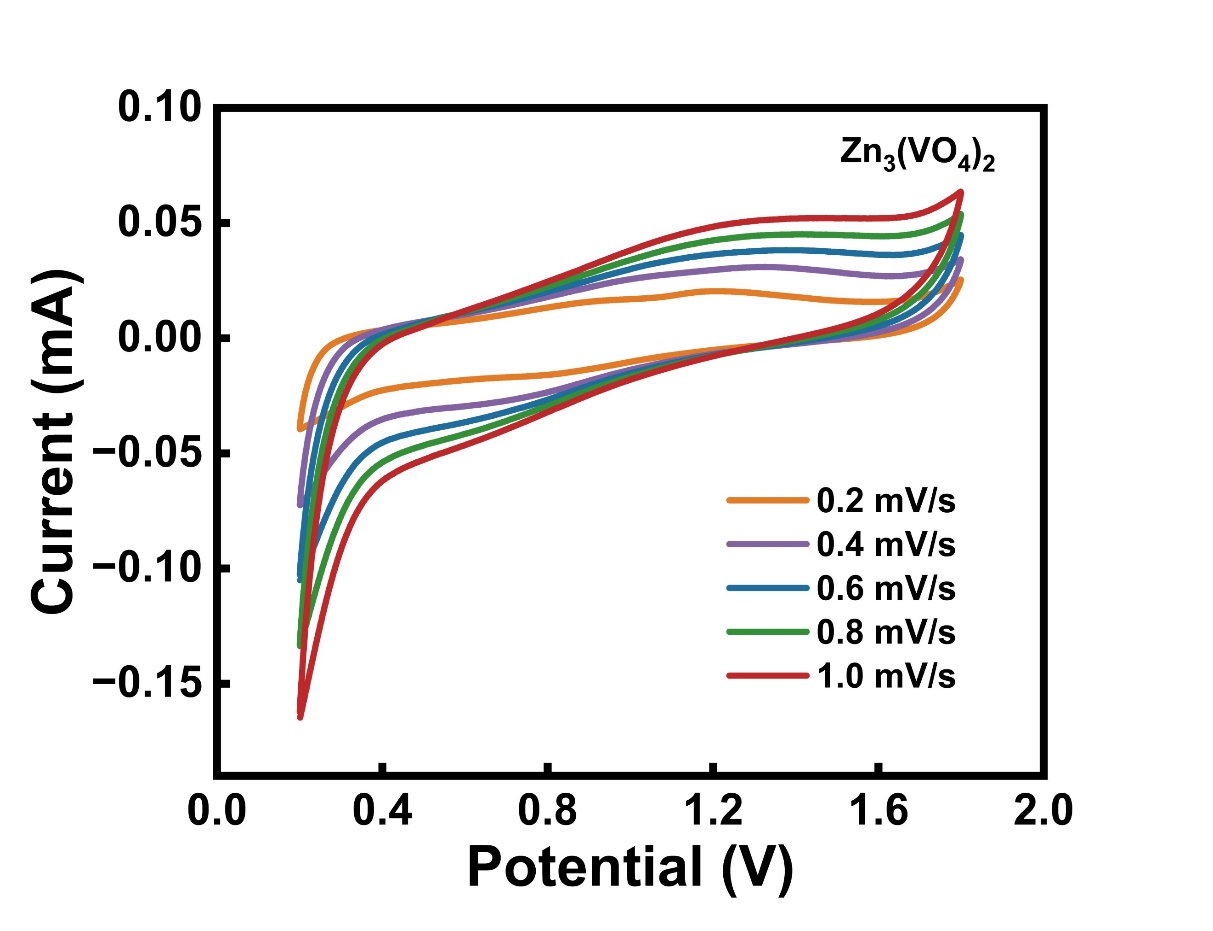


**Figure. S12** CV Curves of Zn_3_(VO_4_)_2_ cathode at different scan rates from 0.2 to 1.0 mV s^-1^.


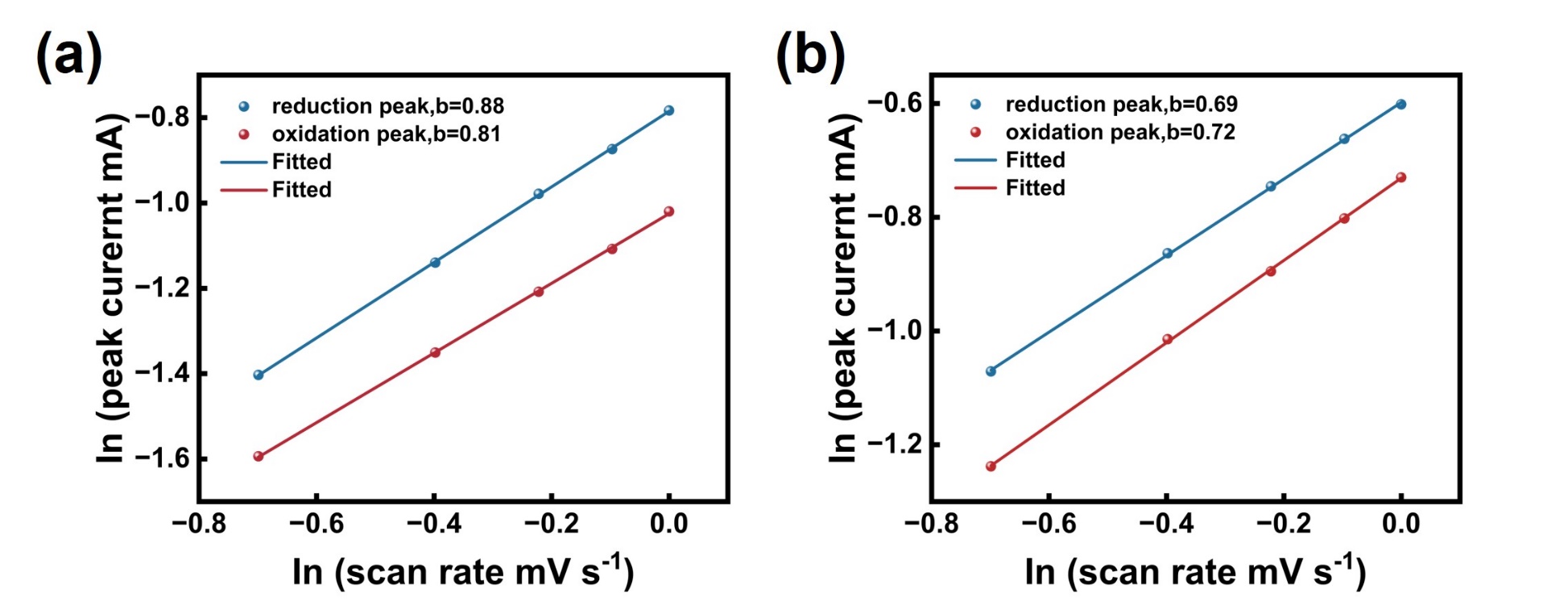


**Figure. S13** The relationship between the peak current of Zn_3_(VO_4_)_2_ and (b) Zn_3_(VO_4_)_2_-0.5V_ZVO_ cathode and the scan rates.


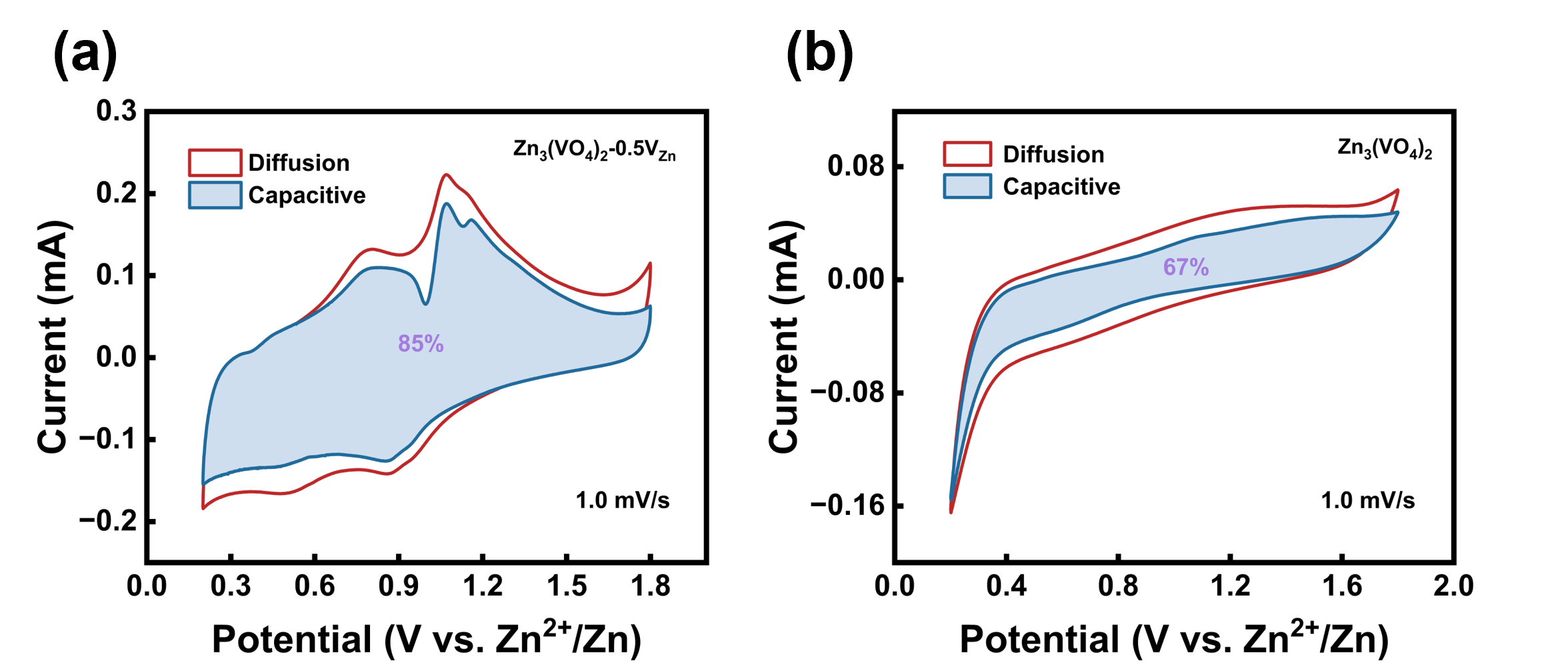


**Figure. S14** The calculated capacitive contribution areas of (a) Zn_3_(VO_4_)_2_-0.5V_ZVO_ and (b) Zn_3_(VO_4_)_2_ cathodes at 1.0 mV s^-1^.


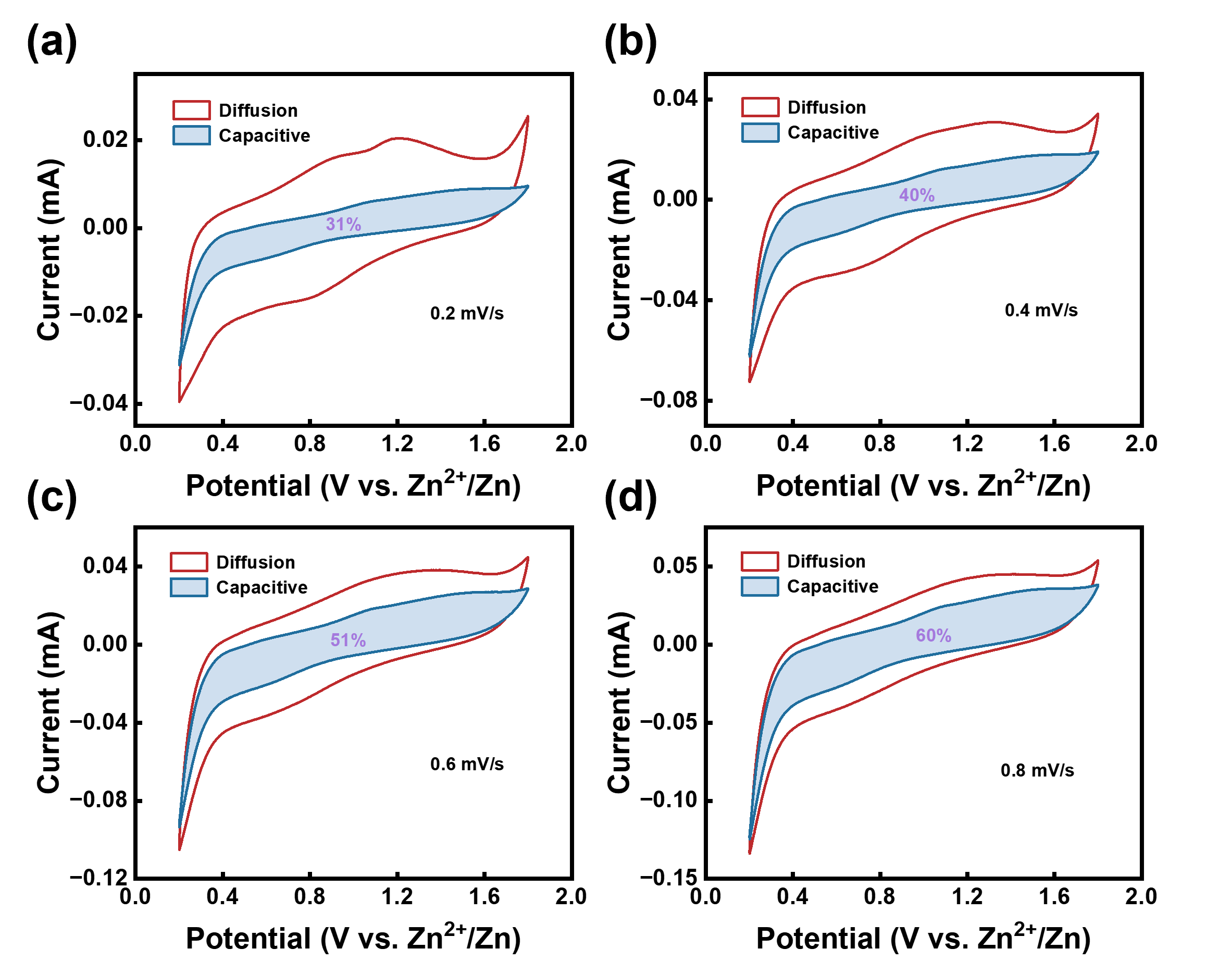


**Figure. S15** The calculated capacitance contribution areas of Zn_3_(VO_4_) cathode at (a) 0.2 mV s^-1^, (b) 0.4 mV s^-1^, (c) 0.6 mV s^-1^, and (d) 0.8 mV s^-1^.


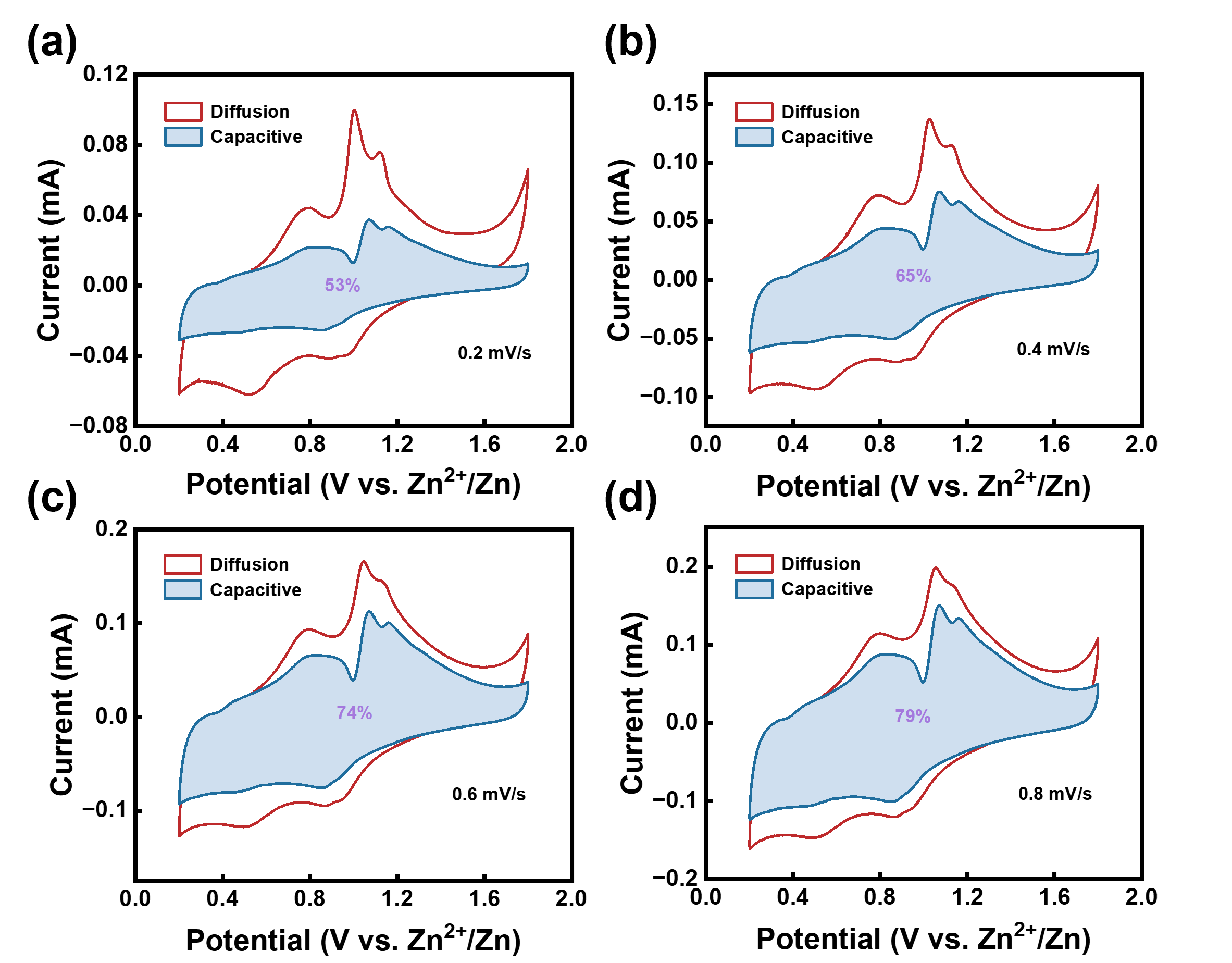


**Figure. S16** The calculated capacitance contribution areas of Zn_3_(VO_4_)_2_-0.5V_ZVO_ cathode at (a) 0.2 mV s^-1^, (b) 0.4 mV s^-1^, (c) 0.6 mV s^-1^, and (d) 0.8 mV s^-1^.


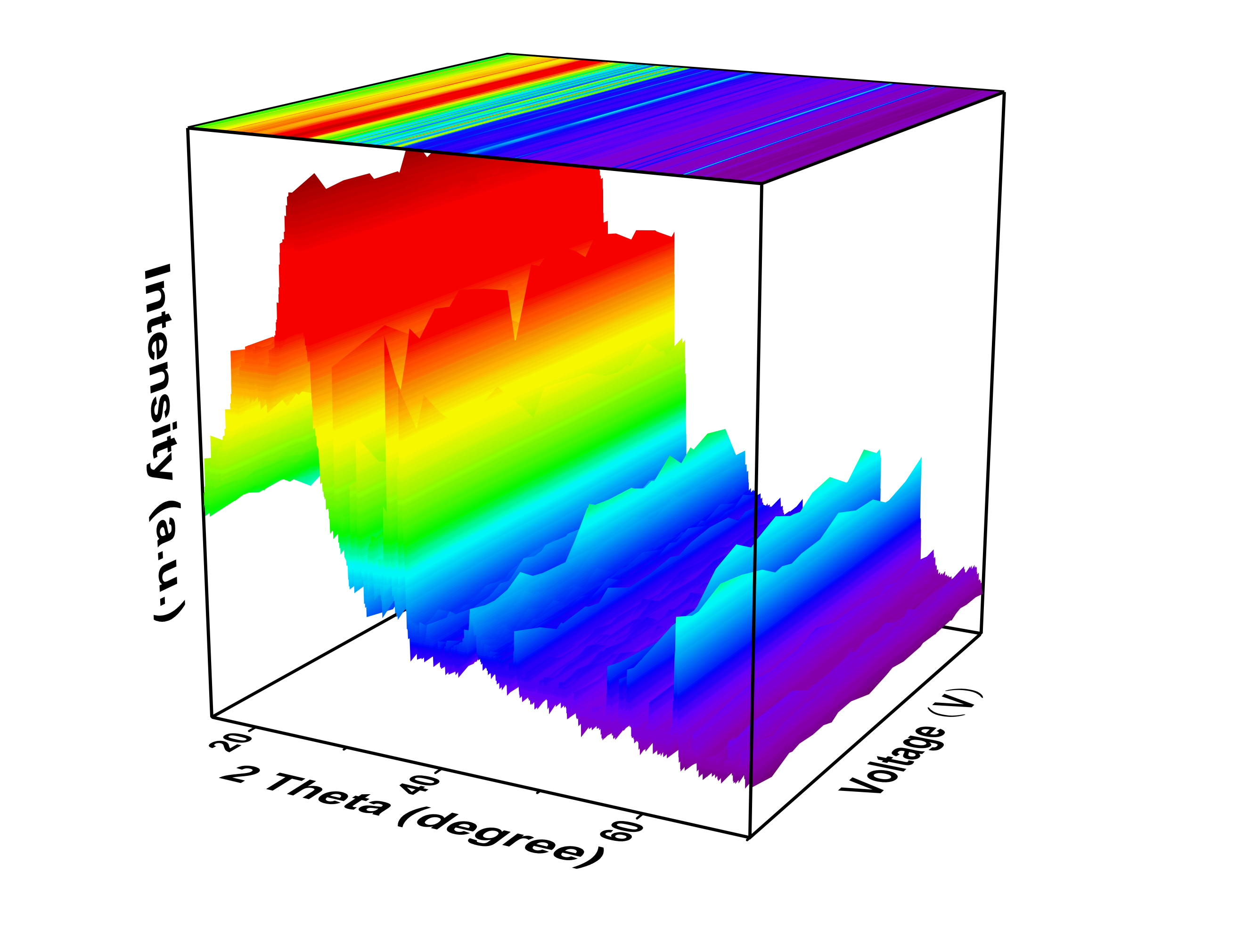


**Figure. S17** Contour map for the *in-situ* XRD patterns in the first cycle of Zn_3_(VO_4_)_2_-0.5V_ZVO_ cathode.

**
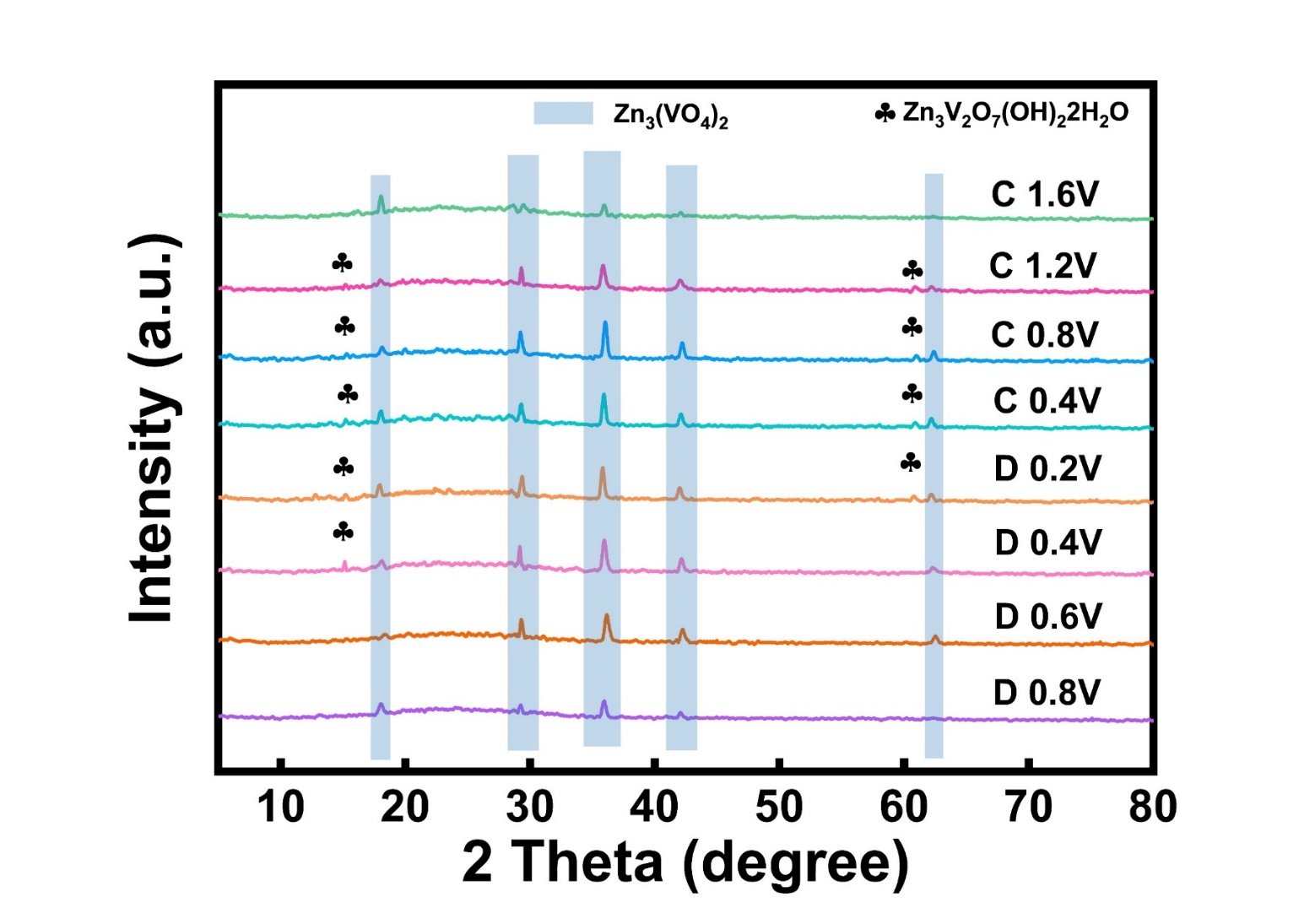
**

**Figure. S18** *Ex-situ* XRD patterns in the second cycle of Zn_3_(VO_4_)_2_-0.5V_ZVO_ cathode.


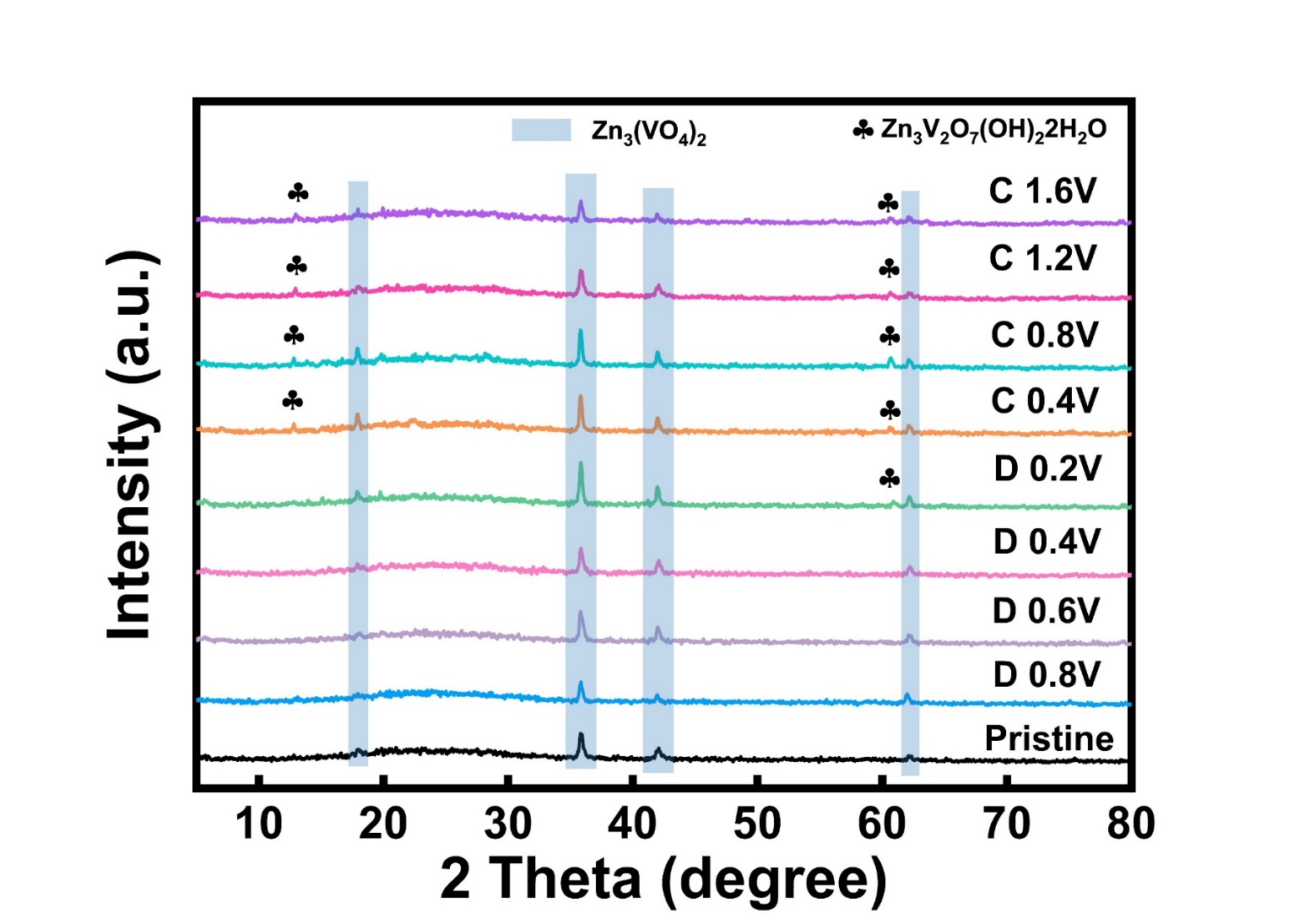


**Figure. S19** *Ex-situ* XRD patterns in the third cycle of Zn_3_(VO_4_)_2_-0.5V_ZVO_ cathode.


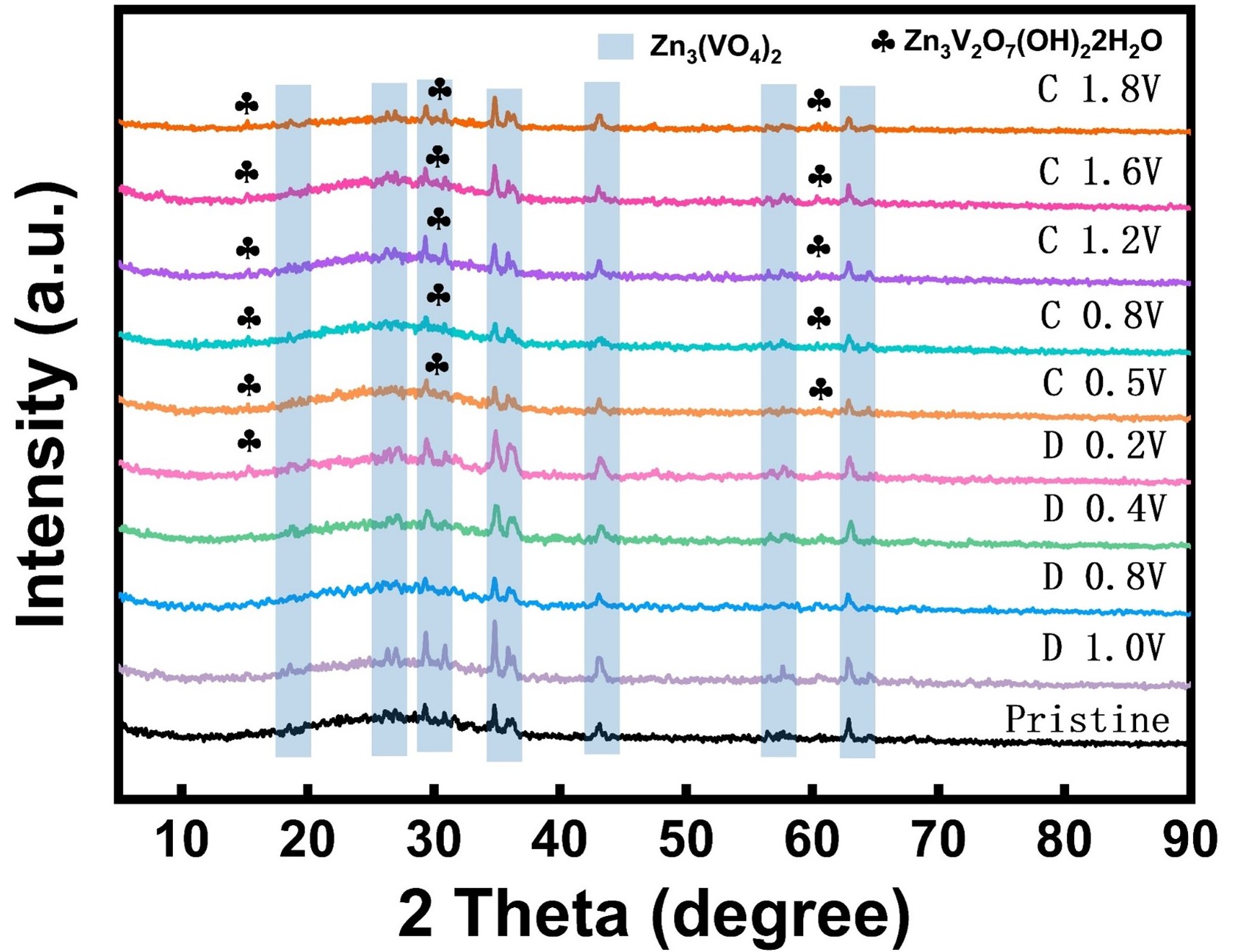


**Figure. S20** *Ex-situ* XRD patterns in the first cycle of Zn_3_(VO_4_)_2_ cathode.

| **Materials** | **Cell parameters prior to adsorption of one Zn** | | | | **Cell parameters after adsorption of one Zn** | | | | **Volume change** |
| --- | --- | --- | --- | --- | --- | --- | --- | --- | --- |
|  | **a (Å)** | **b (Å)** | **c (Å)** | **Volume (****Å)****^3^** | **a (Å)** | **b (Å)** | **c (Å)** | **Volume (Å)^3^** |  |
| Zn_3_(VO_4_)_2_ | 12.22 | 28.94 | 20.64 | 7299.26 | 12.32 | 29.26 | 20.84 | 7512.47 | 2.92 % |
| Zn_3_(VO_4_)_2_-0.5V_zvo_ | 12.35 | 31.51 | 22.46 | 8740.28 | 12.36 | 31.75 | 22.63 | 8880.69 | 1.61 % |

**Table S1** Cell parameters and volume changes before and after Zn adsorption for all calculated models.

**Table S2** Complete benchmarking parameter for comparison of Zn_3_(VO_4_)_2_-V_ZVO_ with typical vanadium-based AZIBs in Figure. S9.

| Sample | Electrolyte | Mass loading (mg cm^-2^) | Cycle number | Current Density  (A g^-1^) | Capacity  (mAh g^-1^) | Reference |
| --- | --- | --- | --- | --- | --- | --- |
| Zn_3_(VO_4_)_2_-V_ZVO_ | 2M ZnSO_4_ | 1 | 40 | 1.0 | 410 | This work |
| K_0.54_V_2_O_5_ | 3M ZnSO_4_ | 1.5-2 | 60 | 1.0 | 209 | ACS Appl. Energy Mater., 2022, 5, 1656-1661. |
| NH_4_V_4_O_10_ | 2M ZnSO_4_ | 1 | 10 | 1.0 | 276 | Energy Storage Mater., 2022, 52, 664-674. |
| (NH_4_)_2_V_10_O_25_·8H_2_O | 2M ZnSO_4_ | 2 | 20 | 1.0 | 240 | Nano Energy , 2021, 90, 106596. |
| (NH_4_)_0.38_V_2_O_5_/CNTs | 2M ZnSO_4_ | 2.1-3.6 | 40 | 1.0 | 281 | Energy Storage Mater., 2021, 42, 286-294. |
| Na_5_V_12_O_32_ | 2M ZnSO_4_ | 2 | 40 | 1.0 | 200 | Adv. Energy Mater., 2018, 8(27), 1801819. |
| α-Zn_2_V_2_O_7_ nanowire | 1M ZnSO_4_ | 3-3.5 | 32 | 1.0 | 210 | J Mater Chem A., 2018, 6(9), 3850. |
| VS_4_ | 1M ZnSO_4_ | **——** | 1000 | 1.0 | 209 | J. Mater Chem. A., 2020, 8, 10761. |
